# Tipping point forecasting in nonstationary dynamics on function spaces

**DOI:** 10.1073/pnas.2600331123

**Published:** 2026-10-15

**Authors:** Miguel Liu-Schiaffini, Clare E. Singer, Nikola Kovachki, Sze Chai Leung, Hyunji Jane Bae, Kamyar Azizzadenesheli, Anima Anandkumar

**Affiliations:** ^a^ Division of Engineering and Applied Science, California Institute of Technology, Pasadena, CA 91125; ^b^ University of Colorado Boulder, Boulder, CO 80309; ^c^ Nvidia Corporation, Santa Clara, CA 95051

**Keywords:** AI, tipping points, neural operators, physics informed learning

## Abstract

Tipping points are abrupt, drastic, and often irreversible changes in the evolution of nonstationary and chaotic dynamical systems. For instance, increased greenhouse gas concentrations are predicted to lead to drastic decreases in low cloud cover, referred to as a climatological tipping point. In this paper, we learn the evolution of such nonstationary dynamical systems using a recurrent neural operator (RNO), which learns mappings between function spaces. After training RNO on only the pre-tipping dynamics, we employ it to detect future tipping points using an uncertainty-based approach. In particular, we propose a conformal prediction framework to forecast tipping points by monitoring deviations from physics constraints (such as conserved quantities and partial differential equations), enabling forecasting of these abrupt changes along with a rigorous measure of uncertainty. We illustrate our proposed methodology on nonstationary ordinary and partial differential equations, such as the Lorenz-63 and Kuramoto–Sivashinsky equations. We also apply our methods to forecast a climate tipping point in stratocumulus cloud cover and airfoil wake and stall transitions using only limited knowledge of the governing equations. For the latter, we show that our proposed method zero-shot generalizes to forecasting multiple future tipping points under varying Reynolds numbers. In our experiments, we demonstrate that even partial or approximate physics constraints can be used to accurately forecast future tipping points.

## Significance

Tipping points are abrupt and often irreversible shifts in complex systems, like the Earth’s climate or fluid flows. We introduce a machine learning framework to forecast future tipping points. While most prior works predict tipping points in simpler systems, we demonstrate that our approach works on large-scale complex problems, including forecasting a climate tipping point in cloud cover and aerodynamic instability in airfoils.

**Fig. 1.**
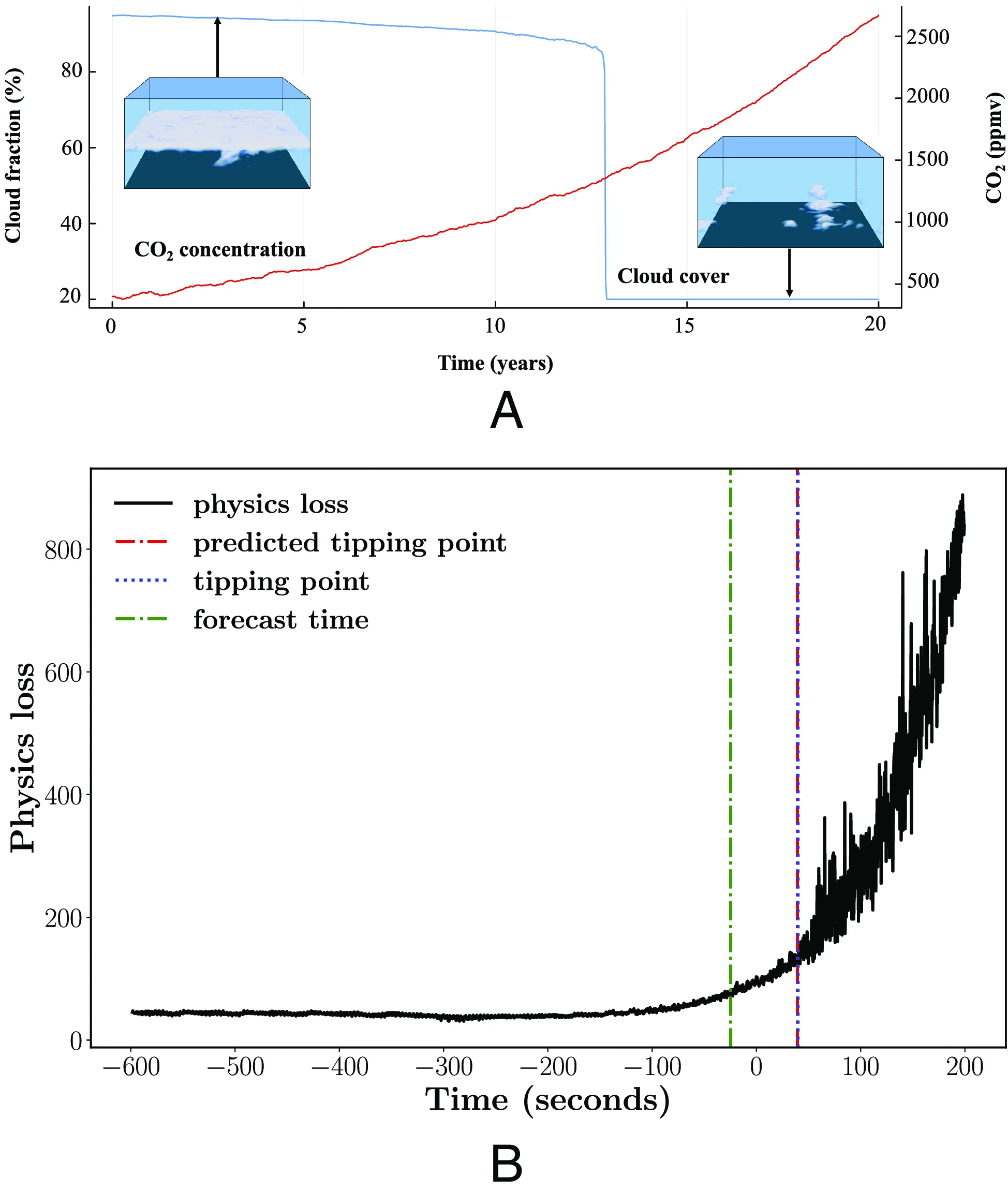
(*A*) Tipping point in cloud fraction as a function of ${\text{CO}}_{2}$ concentration, from bulk model of the atmospheric boundary layer developed in refs. 3 and 4. 3d cloud cover renderings reproduced from ref. 2. (*B*) RNO accurately forecasts tipping point 64 seconds ahead in nonstationary Lorenz-63 system. “Predicted tipping point” is the time at which our framework predicts a tipping point will occur, and “forecast time” is the time when our framework makes this prediction.

Non-stationary chaotic dynamics are a prominent part of the world around us. In chaotic systems, small perturbations in the initial conditions significantly affect the long-term trajectory of the dynamics. Nonstationary chaotic systems possess further complexity due to their time-varying nature. For instance, the atmosphere and ocean dynamics that govern Earth’s climate are modeled by highly nonlinear partial differential equations (PDEs). They exhibit nonstationary chaotic behavior due to changes in anthropogenic greenhouse gas emissions, insolation, and a myriad of complex internal feedbacks (1, 2) (Fig. 1*A*). Additionally, turbulent aerodynamics is governed by the Navier–Stokes equations, a set of nonlinear PDEs that describe the motion of fluids. These equations give rise to chaotic and nonstationary behavior across a wide range of scales, especially in high-Reynolds-number flows commonly encountered in aerospace applications (5). One of the main areas in scientific computing is understanding such phenomena and providing computational methods to model their dynamics. Numerical methods, e.g., finite element and finite difference methods, have been widely used to solve PDEs. However, numerical methods have enormous computational requirements to capture the fine scales in complex processes. Moreover, they do not provide a manageable way to learn from data to reduce scientific modeling errors.

## 1.1. Learning in Nonstationary Physical Systems.

These complexities in modeling complex physical systems make learning the dynamics of their evolution in function spaces notoriously difficult. Prior works proposed various neural networks, such as recurrent neural networks (RNNs) (6) and reservoir computing (7), for learning such dynamical systems (6). However, these methods learn maps between finite-dimensional spaces and are thus not suitable for learning on function spaces, which are inherently infinite-dimensional objects. For example, when modeling spatiotemporal systems, the size of input and output layers in RNNs depend on the number of discretization points in physical space; the parameter count of RNNs depends on the input and output resolutions.

Recent research introduced neural operators (8, 9) for learning operators to remedy these shortcomings. Neural operators receive functions at any discretization as inputs, possess multiple layers of nonlinear integral operators, and output functions that can be evaluated at any point. Neural operators are universal approximators of operators and can approximate continuous nonlinear operators in PDEs (8). Crucially, the number of para meters in neural operators stays constant with respect to the discretization of the inputs and outputs. These properties make neural operators discretization-invariant and suitable for learning in function spaces (10). Fourier neural operators (FNO) are architectures that use Fourier representations to integrate, providing an efficient implementation for many real-world applications. These methods, in particular Markov neural operators (MNO), have shown significant progress in learning Markov kernels of stationary dynamics systems (11). However, nonstationary dynamical systems have potentially long memories, making earlier MNO developments not directly applicable to the setting of this paper.

In this work, we introduce recurrent neural operators (RNOs), a recurrent architecture for neural operators that enables learning memory-equipped maps on function spaces (Fig. 3). RNO receives a sequence of historical continuous (in time) function data and represents the memory in terms of a latent state function. This enables conditional predictions for future functions given the latent functional representations of the past. In contrast to RNNs and other fixed-discretization approaches, RNO is discretization invariant in both space and time. We show that RNOs are capable of learning the dynamics of nonstationary chaotic systems and outperform RNNs and the state-of-the-art MNO by orders of magnitude on a model of stratocumulus cloud cover evolution (3, 4), as well the nonstationary Lorenz-63 system and the Kuramoto–Sivashinsky (KS) equation.

## 1.2. Tipping Point Forecasting.

A commonly observed trait of nonstationarity is the existence of abrupt, drastic, and often irreversible changes in system dynamics, known as tipping points (12). Tipping points are conceptual reference points in nonstationary systems where the evolution undergoes a sudden change in quantities of importance. For instance, in the climate system, several tipping point phenomena have been identified (13–15), such as the collapse of the oceanic thermohaline circulation (16–18), ice sheet instability (19–22), permafrost loss (23, 24), and low cloud cover breakup (2). As shown in Fig. 1*A*, an increase in ${\text{CO}}_{2}$ in an idealized model is linked to a delayed drastic drop in cloud cover (2–4). Another example of tipping points are the wake transition and static aerodynamic stall in a flow over an airfoil with an increasing angle of attack (25, 26).

Predicting tipping points in nonstationary chaotic systems poses significant challenges due to several reasons. First, chaotic systems are highly sensitive to initial conditions, making precise long-term forecasting inherently uncertain. Small variations in initial states can lead to divergent outcomes, complicating the identification of critical thresholds where abrupt transitions occur. Second, the dynamics near tipping points often exhibit complex nonlinear behaviors that defy straightforward analytical or numerical predictions. This complexity arises from the interaction of multiple variables and feedback mechanisms, further complicating the identification of impending transitions. In the context of climate change and airfoil stall, where small changes can lead to large-scale effects such as extreme weather events or loss of lift, accurately pinpointing tipping points is crucial yet remains a formidable scientific and computational challenge.

Identifying and analyzing tipping points in chaotic systems often involves running numerical solvers over extended time horizons, which results in significant computational expense (2, 17). Therefore, solvers are often run at low resolution or over subsets of the full domain, which can result in loss of accuracy or generality (2).

Recently, machine learning methods have been deployed for predicting the tipping points of nonstationary systems (7, 27–37). However, prior works either focus on detecting domain shift instead of forecasting tipping points far ahead in time, and those that do forecast tipping points lack a systematic approach to learn nonstationary systems at arbitrary resolutions and forecast tipping points in function space.

**Fig. 2.**
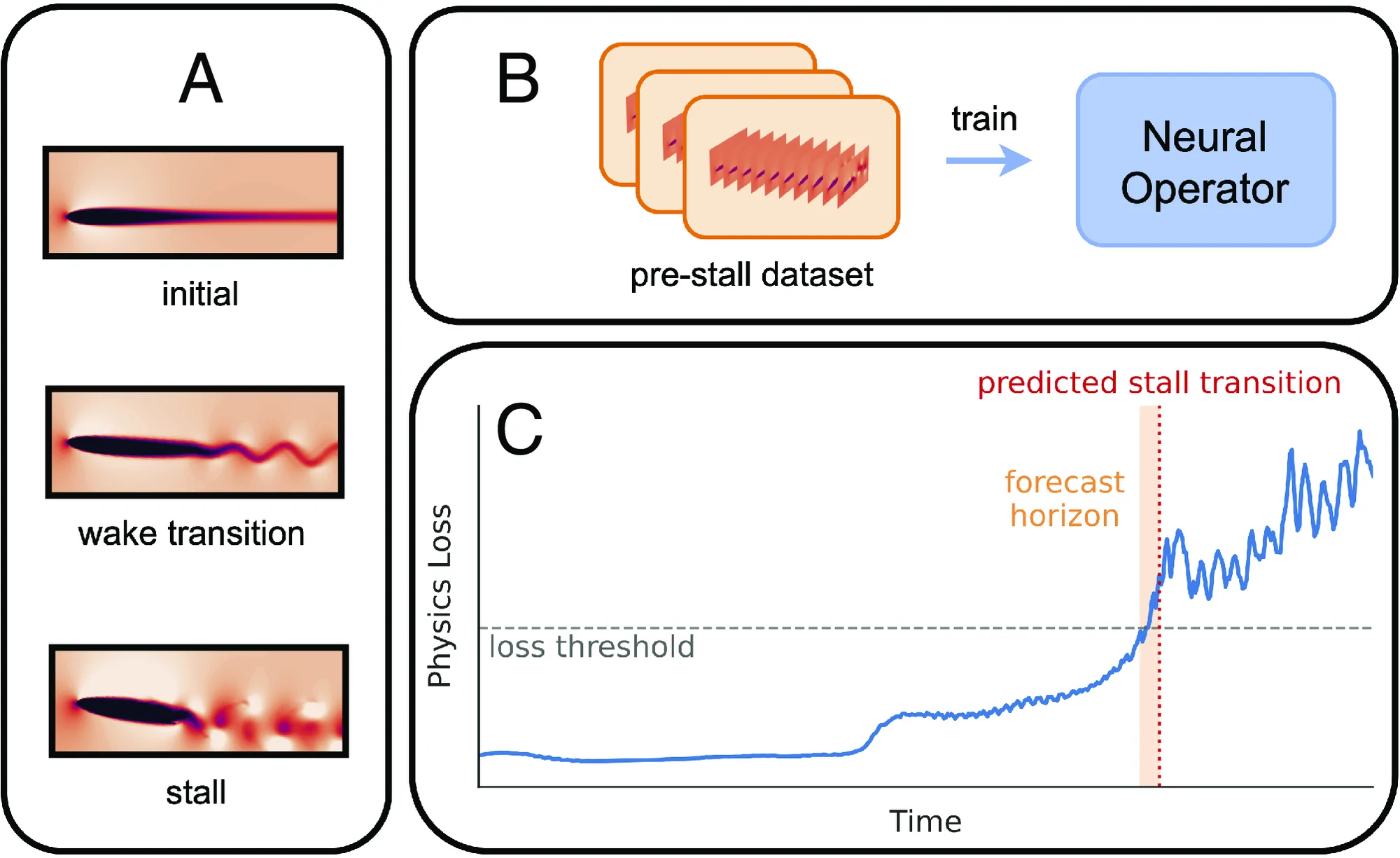
Conceptual diagram of our proposed method applied to a flow over an airfoil with a changing angle of attack. (*A*) The system exhibits two tipping points, the wake and stall transitions. In this example, we consider forecasting the stall transition. (*B*) We train a neural operator model to learn the pre-stall dynamics of the system. Note that our method does not require access to post-tipping data at training time. (*C*) At inference time, we autoregressively roll-out our model’s dynamics predictions and compute the predictions’ physics loss at each time-step. The first time the physics loss crosses a predefined loss threshold, we label this as the tipping point.

In this work, we propose training an RNO model to predict future states on the complex dynamics of nonstationary chaotic systems using only data of pre-tipping point events. The set-up is motivated by climate modeling, where tipping points are not present in forty years of historical data (38). To forecast the time of a tipping point, we build our approach based on the common observation that, at a tipping point, the prediction accuracy of machine learning models degrades due to distribution shifts (7, 28). Since in our setting we do not have access to post-tipping data, we instead propose to forecast tipping points whenever the model prediction of the future exhibits extensive violations of domain-specific physical constraints. We use these physical constraints (e.g., conservation laws or governing PDEs) to verify the correctness of our model’s system forecasts at a given future time. Our method assumes that a given RNO model is well trained on pre-tipping dynamics. Crucially, since the model is trained only on pre-tipping dynamics, even a perfect model on the pre-tipping regime would degrade in prediction accuracy beyond the tipping point, as observed empirically. We show that this approach predicts tipping points very far in advance. See Fig. 2 for a conceptual diagram.

To quantify the prediction accuracy, we propose a conformal prediction method based on cumulative distribution functions (CDFs) (39). We estimate the CDF of physics error, which quantifies the violation of physics constraints by the model’s forecasts and plays the role of a nonconformity score. We employ the fact that using finitely many samples, a CDF of any distribution can be estimated accurately everywhere under the Kolmogorov–Smirnov distance, a property that is not true for other functions like quantiles (40, 41). Using the empirical CDF of the physics error, we quantify the distribution of the nonconformity score. This uncertainty quantification is directly connected to the false positive rate of our prediction approach.

We consider a real-world system describing tipping points in stratocumulus cloud cover (3, 4) and wake and stall tipping points in flow over an airfoil (26). We also consider the more classical nonstationary and chaotic Lorenz-63 and Kuramoto–Sivashinsky (KS) systems. We show that RNO consistently outperforms the state-of-the-art adapted baseline MNO in forecasting tipping points in these systems, particularly for the KS equation. Furthermore, we demonstrate under our proposed approach, RNO is able to accurately forecast tipping points when run for up to 200 time intervals (Fig. 1*B*). Our proposed method can also forecast real-world tipping points, such as the aforementioned cloud cover transition, using only partial knowledge of the underlying physics (Fig. 5*A*). It does so with an error of only 10 d while forecasting nearly 2.5 years in advance.

To demonstrate the scalability of our method to high-dimensional settings, we consider the problem of forecasting the wake and stall transitions in a flow over an airfoil. We find that we are able to successfully forecast the tipping points using limited knowledge of the underlying governing equations (specifically, using only the divergence-free condition) and for various airfoil pitching rates. Further, our method is capable of forecasting the stall tipping point while trained only on pre-wake data, as well as generalizing to forecasting tipping points at a Reynolds number of 5000 while only being trained on Reynolds number 1000 trajectories. We find that our method is also capable of avoiding false positive forecasts when the underlying nonstationary system does not exhibit a tipping point.

In summary, we propose a tipping point forecasting framework that scales to arbitrary tipping points and arbitrary spatiotemporal systems on function spaces. In particular, we

1. Introduce RNO, an operator learning architecture to learn complex nonstationary dynamics, which far outperforms Markovian and RNN baselines in our experiments. For instance, RNO provides up to 70.9% improvement in 20-step autoregressive prediction on the nonstationary Kuramoto–Sivashinsky equation.
2. Construct a tipping point forecasting framework that relies only on 1) a data-driven model of pre-tipping dynamics and 2) a physical constraint of the system.
3. Propose a conformal prediction method to quantify deviations in model physics error in a statistically rigorous manner.
4. Achieve accurate tipping point forecasting far in advance and demonstrate our method’s scalability to spatiotemporal PDEs such as wake and stall transitions in a flow over an airfoil.
5. Show high accuracy in tipping point forecasting using approximate and partial physical constraints, generalizing our method to settings when full physics laws are unknown.
6. Demonstrate high accuracy in zero-shot tipping point forecasting in out-of-domain regimes unseen at training time.

## 2. Related Work

Tipping points exhibit complex behavior, making their study a challenging task in the high-dimensional multimodal setting (1). In recent years, tipping points and their root causes have been categorized for simple systems (such as low-dimensional ODEs), based on stochasticity, rate change, and bifurcations (12, 42, 43). Tipping points can be caused by changes in the condition and sourcing in differential equations, or sometimes, the root cause is the rate at which these conditions change (12, 44). However, in the case of general scientific computing problems on natural phenomena, theoretical studies of tipping points are very challenging, and prior works have primarily focused on their empirical evaluation via numerical simulation.

Tipping points are abundant in scientific computing of climate evolution and control modeling (13–15). Previous work has highlighted the importance of predicting tipping points in the Earth system with the goal of giving early warning ahead of large changes in the climate (45, 46). Approaches have varied depending on the type of tipping behavior considered (47). For instance, it has been shown that changes in atmospheric CO_2_ levels can cause rapid changes in low cloud cover on the Earth (2, 4). And furthermore, after bringing CO_2_ levels below the tipping threshold, these changes may not be readily undone (the system exhibits hysteresis).

Traditional early warning signals (EWS) of tipping points such as increased autocorrelation before tipping events have been studied extensively for many simple systems (46, 48) and for some specific real-world data (49). In the stationary regime, these empirical signals rest on a rigorous mathematical foundation: The statistical properties of systems near critical transitions are characterized through the spectrum (particularly by the spectral gap) of the generator of the transfer semigroup (50–52). Finally, tipping points in the large spatiotemporal systems that motivate this work have not yet been sufficiently studied empirically and theoretically for these indicators to become evident, and for many spatiotemporal systems, thorough empirical evaluation is difficult due to the computational cost of simulations. Thus, data-driven approaches for learning system dynamics and forecasting tipping points is critical for most challenging real-world problems. In *SI Appendix*, section S9.B, we demonstrate that traditional EWS are not reliable at predicting tipping points in the systems we study. Recently, machine learning methods have been deployed for learning in nonstationary dynamical systems and to predict their tipping points (7, 27–37). However, many works require post-tipping data (7, 27), large datasets of labeled time series (34), full governing equations (30), or are not generalizable to arbitrary types of tipping points (27, 32, 36) or large spatiotemporal systems (35). In contrast, our method is scalable, does not need full governing equations, and is not trained on post-tipping data. See Table 1 and *SI Appendix*, section S9.A for a detailed comparison with prior work.

**Table 1.** Comparison of methods

**Method**	**General**	**Func. space**	**Physics**	**Speed**	**Pre-tip**
Solver	✓	✓	×	×	N/A
EWS (45)	×	×	N/A	✓	✓
Bury et al. (27)	×	×	N/A	✓	×
Patel and Ott (7)	✓	×	N/A	✓	×
Ours	✓	✓	✓	✓	✓

“Generality” denotes applicability to arbitrary types of tipping points. “Pre-tip data” denotes whether the method can forecast tipping points using only pre-tipping data.

RNNs are often used to learn in finite-dimensional dynamical systems with memory or in time-series with discrete-time dynamics (6, 53). They consist of blocks of neural networks that carry memory as a finite-dimensional latent state and enable conditional predictions on learned latent historical representations. But since neural networks are maps between finite-dimensional spaces, RNNs are unfit for many scientific computing problems, which often involve *functions* (i.e., infinite-dimensional objects) and their time-evolution. Neural ODE versions of RNNs have similar issues, can only model specific time derivatives, and require expensive solvers for temporal simulation (54–56).

Neural operators are deep learning models that generalize neural networks to maps between function spaces (9, 57, 58), and they are universal approximators of general operators (8). The inputs to neural operators are functions, and the output function can be evaluated at any point in the function domain. These models are thus known to be *discretization invariant* and can take the input function at any resolution. In this paper, we develop the recurrent neural operator (RNO). RNO receives a sequence of historical function data and represents the memory in terms of a latent state function, enabling conditional predictions given learned latent functional representations of the past.

After the initial version of this paper (59) was released, a few related methods were introduced in the literature. Ref. 60 introduce MemNO, an architecture that combines state space models with FNO by introducing memory, and ref. 61 propose a memory-free architecture where the model inputs are short time intervals. Ref. 62 train neural operators autoregressively for better long-time rollouts. Ref. 63 factorize the spatial and temporal dimensions and propose to use structured state space models to model each dimension.

## 3. Materials and Methods

### 3.1. Preliminaries.

A PDE can be seen as a differential law on function spaces. For an input function a from a function space A (e.g., a fluid velocity field), we denote $D_{A}$ as the domain and $R^{d_{A}}$ as the function codomain with dimension $d_{A}$. For any point in the domain $x\in D_{A}$, the function a maps this point to a $d_{A}$-dimensional vector, i.e, $a\left(x\right)\in R^{d_{A}}$ (e.g., the velocity of a fluid at a particular spatial location x). Correspondingly, let U denote the solution or output function space (e.g., the fluid velocity field at a later time), defined on domain $D_{U}$ with codomain $R^{d_{U}}$, i.e., for any function u∈U and any $x\in D_{U}$, we have $u\left(x\right)\in R^{d_{U}}$. For a given a, we define a PDE in generic form as,

[1] $$\begin{array}{c} \begin{array}{cc} L(u,a)(x) & =0,\;\;\text{in}\;D_{U},\\ L^{\prime }\left(u,a\right)\left(x\right) & =0,\;\;\text{in}\;\partial D_{U}, \end{array} \end{array}$$

where L(u,a) is the governing law in the domain $D_{U}$, and $L^{\prime }\left(u,a\right)\left(x\right)$ is the constraint on the domain boundary $\partial D_{U}$. Following the velocity field example, this may be the Navier–Stokes equations with a given set of boundary conditions. A function u is a solution to the above PDE at input a if the function u satisfies both of the PDE constraints (Eq. **1**), comprising an input-solution pair (a,u). We are concerned with learning maps from input function spaces A to output function spaces U in operator learning (e.g., learning the map of a fluid velocity field from time t to t+h). For a given PDE, let $G^{⋆}$ denote the operator that maps the input functions in A to their corresponding solution functions in U, which we seek to learn with a neural operator.

A neural operator G is a deep learning architecture consisting of multiple layers of point-wise and integral operators (8) to learn a map from input function a to output function u. The first layer of the architecture is a pointwise lifting operator $P_{0}$ such that for a given function a, the output of this layer $\nu_{0}=P_{0}a$ is computed so that for any $x\in D_{A}$, $\nu_{0}\left(x\right)=P_{0}\left(a\left(x\right)\right)\in R^{d_{0}}$ where $P_{0}$ is a learnable neural network. This follows standard deep learning architectural practice, where the computation is performed in a higher-dimensional latent space. This step is followed by L layers of nonlinear integral operators. For any layer l we have,

[2] $$\begin{array}{c} \nu_{l}=P_{l}\left(\sigma \left(K_{l}\nu_{l-1}\right)+W_{l}\nu_{l-1}s_{l}\right), \end{array}$$

where $\nu_{l}:D_{l}\to R_{l}^{d}$, σ is some pointwise nonlinearity, and $K_{l}$ is an integral operator such that for any $x\in D_{l}$, we have,

[3] $$\begin{array}{c} K_{l}\nu_{l-1}\left(x\right)=\int_{D_{l-1}}\kappa_{l}\left(x,y\right)\nu_{l-1}d\mu_{l}\left(y\right)+W_{l}^{\prime }\nu_{l-1}\left(s_{l}\left(x\right)\right). \end{array}$$

Here $\kappa_{l}$ is a learnable kernel function, $W_{l}^{\prime }$ is a pointwise operator parameterized by a neural network $W_{l}^{\prime }$, and $s_{l}$ is a deformation map from $D_{l}$ to $D_{l-1}$. Similarly, $W_{l}$ is a pointwise operator parameterized by $W_{l}$ neural network, and $P_{l}$ is a pointwise operator parameterized by a neural network $P_{l}$. $\mu_{l}$ represents the measure on each space and finally, we set $D_{L}=D_{U}$. Intuitively, this kernel integral generalizes matrix multiplication in standard neural networks by integrating over the spatial domain $D_{U}$, where the specific discretization points enter as quadrature in the integral (10). Parameterizing the kernel $\kappa_{l}$ with a finite number of parameters makes the parameter count of a neural network fixed with respect to the spatial discretization. The last layer is a pointwise projection operator Q parameterized by a neural network Q. In particular, we use Fourier representations to compute the integration Eq. **3** in its convolution form,

[4] $$\begin{array}{c} K_{l}\nu_{l-1}\left(x\right)=F^{-1}\left(F\kappa_{l}\cdot F\nu_{l-1}\right)+W_{l}^{\prime }\nu_{l-1}\left(s_{l}\left(x\right)\right), \end{array}$$

where F denotes the Fourier transform and projects its inputs to $k_{l}$ Fourier bases, for some hyperparameter $k_{l}$. This represents the integral operator with a finite number of parameters but still exhibits invariance to the input and output resolutions. Let $R_{l}$ denote the $k_{l}$ Fourier coefficients of $\kappa_{l}$, i.e., $R_{l}=F\kappa_{l}$. Following ref. 57, we directly parameterize $R_{l}$ instead of $\kappa_{l}$ to learn the integral operator. We utilize these building blocks to construct the RNO architecture in the next section.

### 3.2. Recurrent Neural Operator (RNO).

We now describe and formulate RNO, a generalization of RNNs to function spaces. For a given PDE describing the evolution of a function u(x,t) of time and space, consider a partition of the time domain t into equally spaced intervals of length ΔT and indexed by τ∈N. A step τ is associated with the interval [(τ−1)·ΔT,τ·ΔT], where the input function restricted to this interval is defined for t∈[t,t+τ·ΔT] as $u_{\tau }\left(x,t\right):=u\left(x,t\right)$.

Our proposed architecture of the RNO parallels that of the gated recurrent unit (GRU) (64), where the GRU cell at step τ receives a hidden state vector $h_{\tau }$ and an input vector $x_{\tau }$, and predicts the next output vector $x_{\tau +1}$ and next hidden state vector $h_{\tau +1}$, which is also passed to the GRU cell at step τ+1. The key difference between the RNO and the GRU is that at step τ, an RNO cell receives as input a hidden function $h_{\tau }\left(x,t\right)$ (which itself is represented at arbitrary resolutions) and an input function $u_{\tau }\left(x,t\right)$. As such, we replace the linear maps in the GRU architecture with Fourier layers, Eq. **4**). Given $u_{\tau }$ and $h_{\tau }$, we define the reset gate (which intuitively determines what to forget from the past) such that for any t∈[t,t+τ·ΔT],

[5] $$\begin{array}{c} r_{\tau }\left(x,t\right)=\sigma (\left(K_{r}u_{\tau }\right)\left(x,t\right)+\left(K_{r}^{\prime }h_{\tau }\right)\left(x,t\right)+b_{r}\left(x,t\right)), \end{array}$$

where $K_{r},K_{r}^{\prime }$ are Fourier layers, $b_{r}\left(x,t\right)$ is a learned bias function, and σ is the sigmoid function applied pointwise. We define the update gate (which intuitively determines what to remember for the future) as,

[6] $$\begin{array}{c} z_{\tau }\left(x,t\right)=\sigma (\left(K_{z}u_{\tau }\right)\left(x,t\right)+\left(K_{z}^{\prime }h_{\tau }\right)\left(x,t\right)+b_{z}\left(x,t\right)). \end{array}$$

We define the candidate hidden function as,

[7] $$\begin{array}{c} {\hat{h}}_{\tau +1}\left(x,t\right)=\phi (\left(K_{h}u_{\tau }\right)\left(x,t\right)+\left(K_{h}^{\prime }\left(r_{\tau }\cdot h_{\tau }\right)\right)\left(x,t\right)+b_{h}\left(x,t\right)), \end{array}$$

where ϕ denotes a point-wise scaled exponential linear unit (SELU) activation and the function multiplication (·) is taken to be pointwise. Finally, we define the hidden state function $h_{\tau +1}\left(x,t\right)$ as

[8] $$\begin{array}{c} h_{\tau +1}\left(x,t\right)=(\left(1-z_{\tau }\right)\cdot h_{\tau })\left(x,t\right)+(z_{\tau }\cdot {\hat{h}}_{\tau +1})\left(x,t\right). \end{array}$$

We take $h_{0}\left(x,t\right)$ to be a learned prior function on the initial hidden state. Let $\text{RNO}(v,h_{\tau })$ denote an RNO cell. Given the input function $u_{\tau }$, an RNO block takes the following form:

[9] $$\begin{array}{cc} {\tilde{u}}_{\tau }\left(x,t\right) & =P_{0}\left(u_{\tau }\right)\left(x,t\right)=P_{0}\left(u_{\tau }\left(x,t\right)\right), \end{array}$$

[10] $$\begin{array}{cc} v_{\tau }^{\left(i+1\right)}\left(x,t\right) & =\;{\text{RNO}}^{\left(i+1\right)}\left(v_{\tau }^{\left(i\right)},h_{\tau }^{\left(i+1\right)}\right)\left(x,t\right), \end{array}$$

**Fig. 3.**
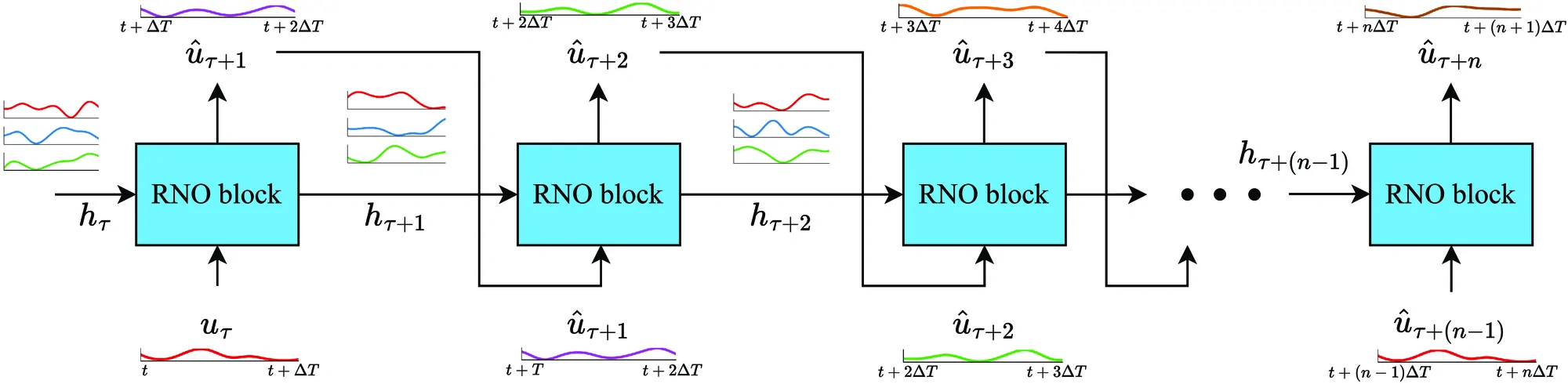
Illustration of RNO autoregressive forecasting.

[11] $$\begin{array}{cc} {\hat{u}}_{\tau }\left(x,t\right) & =Q\left(v_{\tau }^{\left(L\right)}\right)\left(x,t\right)=Q\left(v_{\tau }^{\left(L\right)}\left(x,t\right)\right), \end{array}$$

where $P_{0}$ and Q are pointwise lifting and projection operators, respectively parameterized by neural networks $P_{0}$ and Q. $v_{\tau }^{\left(0\right)}\left(x,t\right):={\tilde{u}}_{\tau }\left(x,t\right)$, the result of the lifting operation, and L is the number of RNO layers. An RNO is then a RNO block evolving in time with $h_{\tau +1}^{\left(i\right)}:=v_{\tau }^{\left(i\right)}$ for each RNO cell i≥1, see Fig. 3.

We divide the inference stage of RNO into two phases:

1. **Warm-up phase:** In the warm-up phase, we have access to the ground-truth trajectory between steps τ=0 and $\tau =N_{\tau }-1$, corresponding to the ground-truth solution u(t) between times $t\in [0,N_{\tau }\Delta T]$. During this phase, the RNO blocks receive as input $u_{\tau }$, and the predicted ${\hat{u}}_{\tau +1}$ is discarded.
2. **Prediction phase:** In the prediction phase, we assume we no longer have access to the ground-truth trajectory beyond time $\tau =N_{\tau }-1$, so the model predictions ${\hat{u}}_{\tau +j}$ are taken as input to the RNO block at time τ+j+1, and the model is thus composed with itself (with access to its hidden state $h_{\tau }$), with its outputs fed back on itself as inputs.

The prediction phase is similar to Markov neural operator (MNO) (11), with the exception that RNO now has access to a continually updating hidden representation $h_{\tau }\left(x,t\right)$, which encodes the history of the nonstationary system, making RNO a generalization of MNO to systems with memory.

### 3.3. Tipping Point Forecasting.

Forecasting tipping points requires rigorous uncertainty quantification, and any prediction must be accompanied by the event’s potential probabilities. Conformal prediction is a field of study that accompanies predictions with certainty levels in terms of a conformity score shafer2008tutorial. Most prior work on traditional conformal prediction quantifies the probability distribution of the conformity score at certain levels. Another approach is to utilize quantile regression to quantify the distribution of conformity scores (65). Both of these approaches have shortcomings in tipping point prediction. The former approach utilizes exchangeability and requires new and fresh draws of samples to quantify the distribution of conformity scores, imposing a union bound over a long period of time. Quantile regression suffers from two downsides: 1) quantiles are not generally estimable, and 2) a union bound over an infinite set is required to quantify the conformity score distribution (66). These limitations are addressed below by introducing CDF estimation methods.

Our tipping point forecasting utilizes training on the pre-tipping dynamics of the system. We train RNO on datasets of time-evolving functions that do not contain any tipping points. We make this choice to resemble real-world settings such as climate where recorded historical data does not contain tipping points. At any time t, we use RNO to predict the long evolution of the dynamics for an interval of [t,t+T], i.e., $\hat{u}\left(x,\left[t,t+T\right]\right)$ where T is a multiple of ΔT. At the forecast time t, we do *not* have access to the future u(x,[t,t+T]). If we did, we could use the deviation of $\hat{u}\left(x,\left[t,t+T\right]\right)$ from u(x,[t,t+T]) as a hindsight indicator for the tipping point as observed in prior works (7, 28).

Instead, we propose to use the physical constraints L(u)=0 of the underlying system (such as the PDE that governs the dynamics) to evaluate the deviation of $\hat{u}\left(x,\left[t,t+T\right]\right)$ from physics laws, and we use this signal as an indicator of tipping points. For a predicted function $\hat{u}\left(x,\left[t,t+T\right]\right)$, we define the conformity score as $\left\|L(\hat{u})-0\right\|$, and we refer to it by the physics loss $\ell_{P}\left(\hat{u}\right)$. Note that if $\hat{u}\left(x,\left[t,t+T\right]\right)=u\left(x,\left[t,t+T\right]\right)$ (the true future function), then $L(\hat{u})=0$, and thus our proposed indicator aligns with the hindsight indicator mentioned above. At any time t, we compute $\ell_{P}\left(\hat{u}\right)$, and if it takes values above a certain threshold, we forecast that a tipping point is anticipated. This method enables tipping point forecasting. However, since the trained model comes with training generalization error, this approach makes mistakes, confusing large errors on pre-tipping regimes and actual tipping incidence. This is of particular importance when the forecasting interval of interest T is large. We propose the following conformal prediction to address uncertainty quantification in tipping point forecasting. For a trained model and given a forecasting interval T, let F denote the true CDF of the conformity score. The CDF captures the probabilities at which the model makes errors of a given magnitude on pre-tipping data. We use the CDF to develop our conformal prediction method, which allows for uncertainty quantification of tipping point forecasts. In particular, for any loss threshold at which we call a tipping point, we can denote α to be the probability of falsely forecasting a tipping point when the data was simply from the pre-tipping regime. We first compute the empirical CDF $\hat{F}$ of the conformity score $\ell_{P}\left(u\right)$ using n calibration samples. Formally, given n samples, we construct $\{{\ell_{P}}^{\left(j\right)}{\}}_{j=1}^{n}$ and compute $\hat{F}$ as follows. For any p∈R,

$$\begin{array}{c} \hat{F}\left(p\right):=\frac{1}{n}\sum_{j}^{n}1\left(p\geq {\ell_{P}}^{\left(j\right)}\right). \end{array}$$

**Fig. 4.**
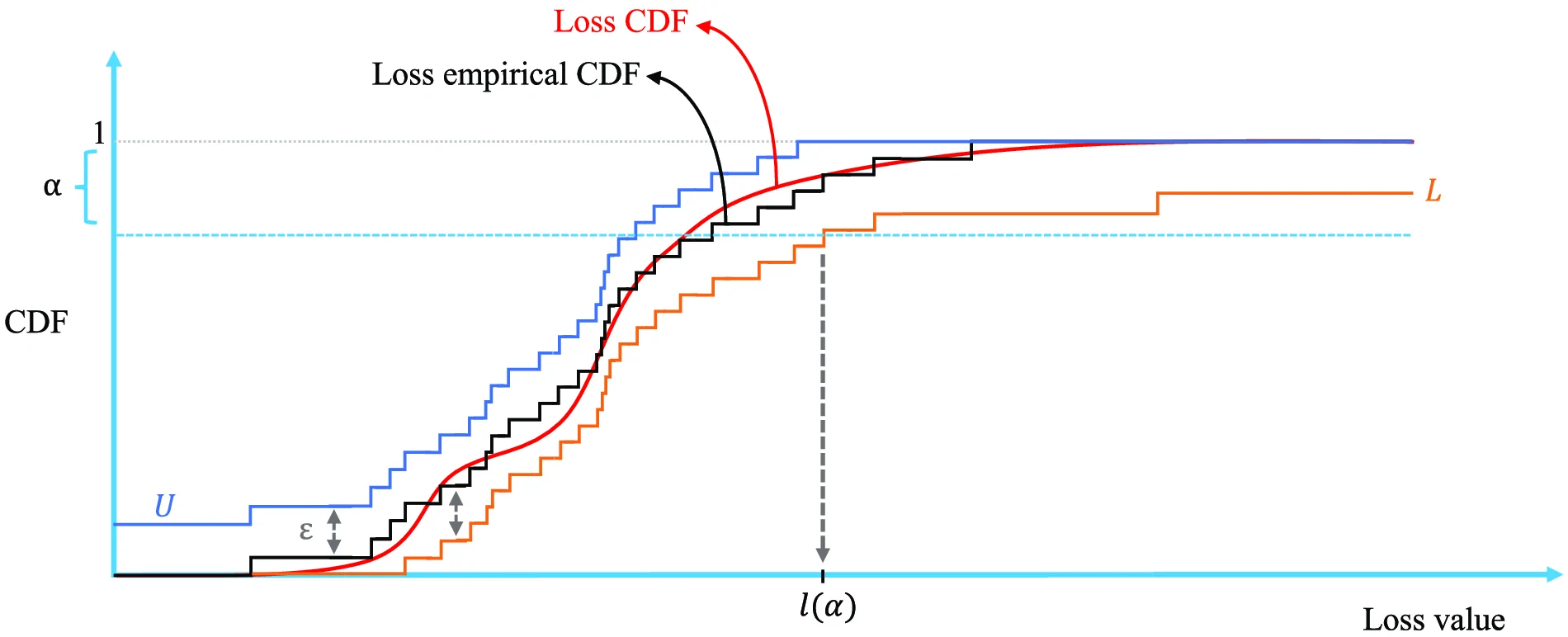
Diagram of the setup for the tipping point prediction method. U and L correspond to the upper and lower bounds given by the CDF concentration inequality, differing from the empirical CDF by ε. α is the significance level, and l(α) is the corresponding loss of L at α.

We then use the concentration inequality of CDFs to compute its upper and lower bounds (40, 41). Using the concentration inequality of CDFs, with probability at least 1−δ we have,

$$\begin{array}{c} \underset{p\in R}{\sup}\left|\hat{F}\left(p\right)-F\left(p\right)\right|\leq \varepsilon :=\sqrt{\frac{\log(2/\delta )}{2n}}, \end{array}$$

where the left-hand side is the Kolmogorov–Smirnov distance between the two CDFs. We define the CDF lower bound $L\left(p\right):=\max\{0,\hat{F}\left(p\right)-\varepsilon \}$ and the CDF upper bound $U\left(p\right):=\min\{1,\hat{F}\left(p\right)+\varepsilon \}$. Fig. 4 depicts this procedure. Intuitively, α denotes the maximum probability of a mistake that one is willing to tolerate. In our approach, as shown in Fig. 4, we first find l(α), the conformity level at which the CDF lower bound L has probability 1−α. Using the level l(α), we call the presence of a tipping point whenever the conformity score of our prediction $\ell_{P}\left(\hat{u}\right)$ is above l(α).

Proposition 1. *For any given* α∈[0,1], *the decision rule of calling for a tipping point at level* l(α) *has a false positive rate of at most* α, *and this statement holds with probability at least* 1−δ.

The proof is based on the fact that for any α, $F^{-1}\left(1-\alpha \right)\leq l\left(\alpha \right)$ and F(l(α))≥1−α. Therefore, when $\ell_{P}\geq l\left(\alpha \right)$, signaling a tipping point, we also have $\ell_{P}\geq F^{-1}\left(1-\alpha \right)$. Thus, the probability of the event $\ell_{P}\geq l\left(\alpha \right)$ is lower bounded by the probability of $\ell_{P}\geq F^{-1}\left(1-\alpha \right)$ which at most α. This proposition simultaneously holds for all the time steps τ and all α. This property enables assessing tipping predicting at various levels, a crucial feature for risk assessment and policy making.

## 4. Results

We present experimental results on learning dynamics and forecasting tipping points in nonstationary systems. We showcase the performance of RNO and the tipping point forecasting method on a simplified model of cloud cover equations and on predicting the turbulent wake and stall tipping points in a 2-d flow over an airfoil. In *SI Appendix*, section S1, we also present detailed results for the finite-dimensional Lorenz-63 system, and for the (infinite-dimensional) KS PDE in *SI Appendix*, section S2. For Lorenz-63, we show that our method is capable of forecasting tipping points far in advance *using only approximate knowledge of the underlying system*. In *SI Appendix*, section S7, we show that our method is robust to different rates of parameter change and can avoid predicting a tipping point when the underlying system lacks one. For the nonstationary KS equation, we show that RNO far outperforms MNO and RNN in state prediction for rollouts of various lengths. Discussion on the technical details of RNO (e.g., memory usage, inference times, hyperparameter selection, etc.) can be found in *SI Appendix*, section S10.

### 4.1. Learning Nonstationary Dynamics.

We compare RNO against Markov neural operators (MNO) (11) and RNNs in forecasting nonstationary dynamics. There are two key properties that any learned model must exhibit: 1) low step-wise error and 2) error stability in long-time predictions (i.e., when the model is composed with itself many times). Low step-wise error is a necessary prerequisite to adequately learning the dynamics of any system. However, for many downstream tasks of significant scientific importance, it is also crucial that our model’s error remain low when forecasting far into the future. This ensures the accuracy of tipping point prediction is credible in complex real-world settings. Hereafter, we define “n-step prediction” to be a model’s forecasting prediction looking ahead τ=n steps.

In our experiments, we employ a multistep training regimen for all models, where the total loss is a combination of the loss on the model’s n-step prediction for n∈{1,2,⋯,M}. That is, our data loss function is given by

[12] $$\begin{array}{c} \ell_{D}\left(u,\hat{u}\right)=\sum_{n=1}^{M}\lambda_{n}{\left\|u_{n}-{\hat{u}}_{n}\right\|}_{2}, \end{array}$$

where ${\left\|\cdot \right\|}_{2}$ is the $L^{2}$ norm and $\lambda_{n}$ is some scaling hyperparameter.

Table 2 compares the relative $L^{2}$ errors of RNO, MNO, and RNN on forecasting the nonstationary KS equation (setup and details in *SI Appendix*, section S2). We observe that RNO outperforms MNO and RNN on every forecasting interval. Further, we can observe the disadvantages of RNN and other finite-dimensional, fixed-resolution models on learning in PDE (i.e., infinite-dimensional) settings. The RNN’s error is orders of magnitude larger than that of RNO or MNO for small forecasting windows. At large forecasting windows (i.e., 12-step and above), the lack of explicit history in MNO becomes a severe disadvantage. In contrast, RNO maintains much lower error at large forecasting intervals than the other two models. Both the MNO and RNO shown in Table 2 were trained with M=5 steps of fine-tuning (Eq. **12**). Observe that MNO and RNN have difficulty generalizing to forecasting intervals larger than M, whereas RNO is capable of maintaining stable error even at longer forecasting intervals.

**Table 2.** Relative $L^{2}$ errors on the nonstationary KS equation for different n-step prediction settings

Model	1-step	2-step	4-step	8-step	12-step	16-step	20-step
RNO	0.0030	0.0027	0.0031	0.0419	0.1410	0.2919	0.4008
MNO	0.0050	0.0073	0.0150	0.2901	1.0751	1.2626	1.3772
RNN	0.0525	0.0481	0.1443	0.5488	0.9031	1.0031	1.1561

RNO outperforms both MNO and the baseline RNN on every forecasting intervals.

### 4.2. Cloud Cover Equations: Tipping Points from Partial Physics.

We now consider a case study of tipping point forecasting in the Earth’s climate. We also demonstrate that our method is still accurate under partial physics, e.g., conservation laws (not the full governing equations). We use the ODE model developed in refs. 3 and 4 as an idealization of the physical climate system; this model exhibits a tipping point. The model represents a stratocumulus-topped atmospheric boundary layer, which experiences rapid loss of cloud cover under high CO_2_ concentrations and shows hysteresis behavior, where the original state is not immediately recovered when CO_2_ concentrations are lowered. This model was developed, in part, to help explain the results found from the high-resolution simulations in refs. 2 and 67. More details on the model can be found in *SI Appendix*, section S3, and details on numerical experiments can be found in *SI Appendix*, section S8.D.

We train an RNO to forecast the nonstationary evolution of the system with a data loss given by Eq. **12**, which incorporates the $L^{2}$ loss across longer time horizons. We compute the empirical CDF of the physics loss for the cloud cover system (analogous to *SI Appendix*, Eq. **S5** in the KS setting) as a function of model predictions and CO_2_. However, in this case, the physics loss is normalized component-wise by the state of the system at a given time, since the magnitudes of each component in this model differ by several orders.

**Fig. 5.**
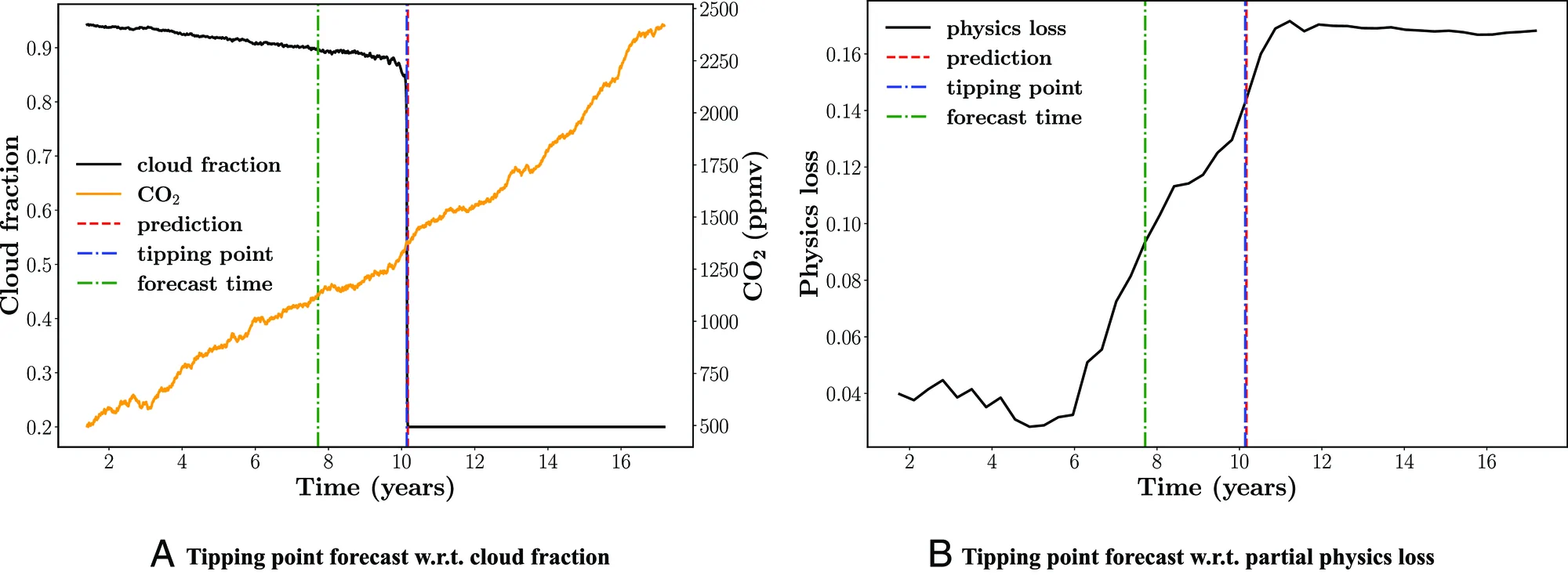
For the cloud cover model, (*A*) appropriately stylized, (*B*) shows the tipping point forecasting time as a function of the physics loss, using only a mass conservation constraint (as opposed to the full system), RNO is still capable of identifying the true tipping point of the system with an error of 0.03 years, predicting T=2.45 years ahead, at α=0.07.

For tipping point forecasting, we base the physics loss solely on mass conservation (*SI Appendix*, Eq. **S6a**), instead of the full ODE. Specifically, we let $\ell_{P}$ be the $L^{2}$ norm of the difference between the left and right-hand-sides of Eq. S6a, which are both functions of time within a time interval. In our experiments, we set the tipping point forecasting horizon to be T=7·ΔT=2.45 years (with forecasting intervals of ΔT=0.36, or 128 d). We found that the choice of ΔT (on time-scales on the order of O(100) days) does not significantly impact the RNO’s ability to learn the dynamics. T is chosen to be an order of magnitude larger than ΔT, to demonstrate that our framework is capable of forecasting tipping points at time-scales much longer than ΔT. The results on a given test trajectory can be seen in Fig. 5. Despite the abruptness of the tipping point and the highly nonlinear dynamics of the system, our forecasting framework is successfully able to identify the true tipping point in this system using a physics loss derived from a partial picture of the full physics.

### 4.3. Forecasting Airfoil Wake and Stall Transitions.

To demonstrate the scalability of our method to complex higher-dimensional settings, we consider the problem of forecasting two tipping points in a 2-d flow over an airfoil with angle of attack that increases at a constant rate over time. We simulated the flow past an airfoil of chord length C with velocity $U_{\infty }$ by solving the 2-d incompressible Navier–Stokes equation using a large-eddy simulation (LES) solver (more details can be found in *SI Appendix*, section S4 and in ref. 68). Two distinct tipping points in the lift coefficients can be observed as a function of the angle of attack (alternatively, as a function of time, since the angle of attack increases at a constant rate over time). The first tipping point observable in the lift coefficient is known as the critical bifurcation from singular-vortex shedding to vortex-pair shedding, which we refer to simply as the wake transition (25). The second tipping point corresponds to the static aerodynamic stall, which is characterized by a rapid drop in the lift coefficient for a small increase in the angle of attack (26). At low Reynolds numbers (Re), however, this drop tends to occur more gradually at angles of attack around 15 degrees (69).

In our experiments, we consider three settings: 1) a Re of 1000, where only the static stall occurs, 2) the wake transition in a flow with Re 5000, and 3) static stall in a flow with Re 5000. Creating these three setups allows us to create a direct progression in the difficulty of each subtask. For instance, we would expect a priori that forecasting 1) is easier than forecasting 2) or 3) because the Re 1000 dynamics are less turbulent than the Re 5000 dynamics. Similarly, we would a priori expect that 3) is more challenging than 2) because it requires forecasting one tipping point after another and thus would require a model that has learned dynamics under multiple regimes.

We first train three separate RNO models to forecast the nonstationary pre-tipping evolution of systems 1), 2), and 3) using the data loss Eq. **12**. The tipping point forecasting methodology is identical to Section 4.2, except the physics loss is now defined by the divergence-free condition of incompressible Navier–Stokes $\ell_{P}\left(u\right)={\left\|\nabla \cdot u\right\|}_{2}$, where u is the velocity field. We note that this physical constraint assumes only minimal physical knowledge of the system.

In our experiments, we also compare the tipping point forecasting performance using RNO, MNO, and RNN. The full results for these settings can be found in *SI Appendix*, section S6, and experiments exploring robustness to different rates of change of the airfoil angle of attack, as well as nontipping settings, can be found in *SI Appendix*, section S7. We find that RNO produces the most accurate forecasts for each setting, over various choices of α. However, MNO and RNN are often still able to forecast the transitions, suggesting that our method is robust to the quality of the temporal evolution model.

**Fig. 6.**
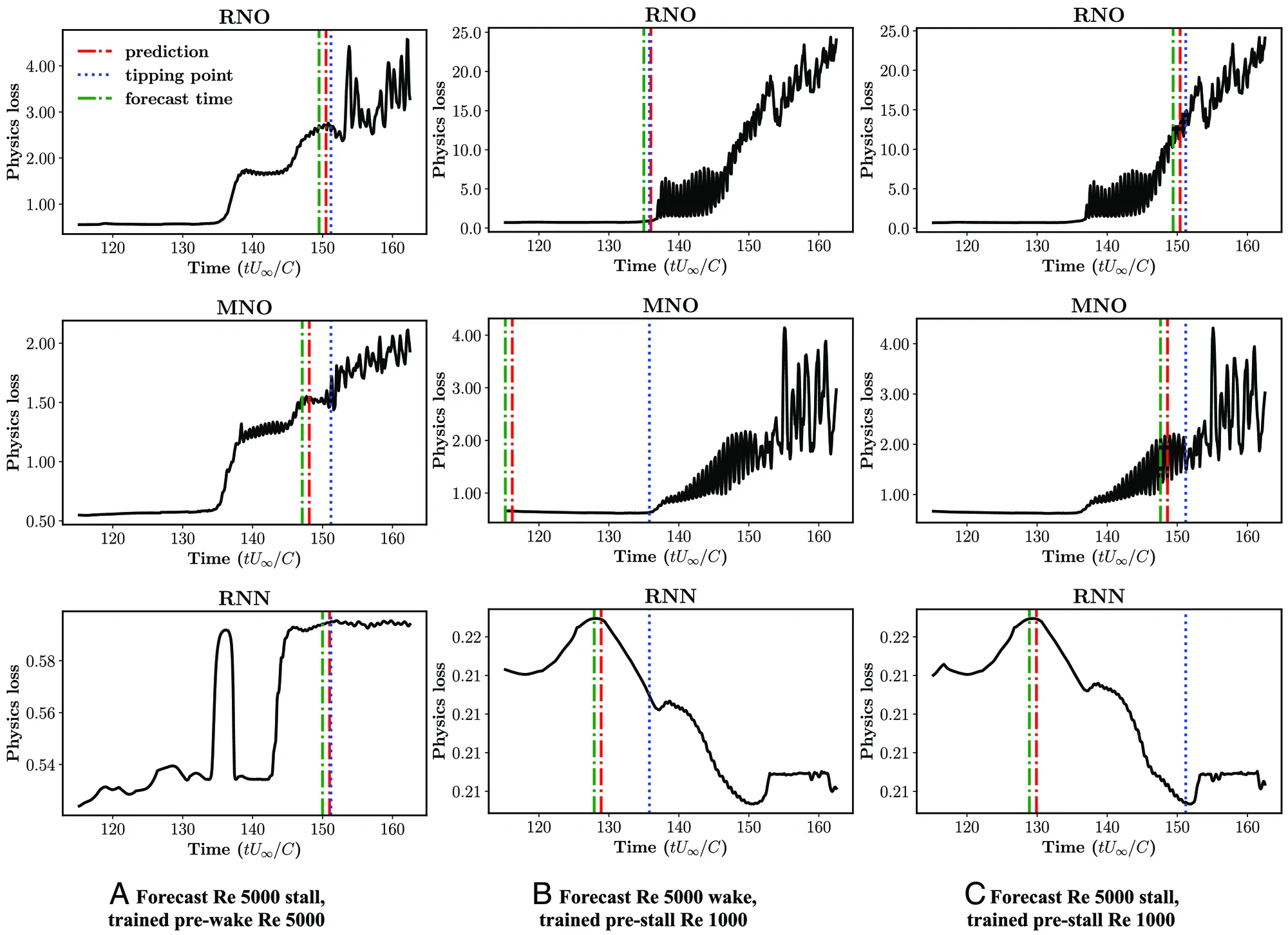
Tipping point forecast comparison between RNO, MNO, and RNN in three generalization settings for the flow over the airfoil setting. (*A*) considers forecasting the stall transition in a Re 5000 flow with the dynamics model trained on pre-wake Re 5000 data. (*B*) considers forecasting the wake transition in a Re 5000 flow with the dynamics model trained on pre-stall Re 1000 data. (*C*) considers forecasting the stall transition in a Re 5000 flow with the dynamics model trained on pre-stall Re 1000 data. (*A* and *C*) use α=0.11, and (*B*) uses α=0.14.

These results motivate the evaluation of the generalizability of our methodology to future tipping points. Fig. 6*A* shows the results of the RNO, MNO, and RNN trained on pre-wake data for Re 5000 but used to forecast the stall tipping point for Re 5000 at inference time. Once again, we observe that the stall point is mostly correctly forecasted by all three models.

Last, we evaluate the performance of our methods on generalizing to forecasting tipping points at different Reynolds numbers. Specifically, we train each of the three models on Re 1000 pre-stall data and use these models to forecast the wake and stall tipping points in the Re 5000 setting. Fig. 6*B* shows the results of RNO, MNO, and RNN trained on Re 1000 pre-stall dynamics and used directly to forecast the Re 5000 wake tipping point. Fig. 6*C* shows the analogous results for the Re 5000 stall tipping point. The results show that RNO far outperforms MNO and RNN in forecasting the tipping points. This suggests that the generalization ability of our tipping point forecasting method depends on the accuracy of the underlying dynamics model.

### 4.4. Discussion.

From the experiments above (and those in the appendix), we can extract several key takeaways about our proposed tipping point forecasting method:

1. Tipping point forecasting remains robust under approximate physical constraints (*SI Appendix*, section S1.C).
2. Tipping point forecasting remains robust using a physics loss based on partial physics laws (i.e., not the full governing equations) and conservation laws (Sections 4.2 and 4.3).
3. Our proposed method is easily applicable to large-scale systems with complex tipping points, even when knowledge of the full governing equations is not known (Section 4.3).
4. Our proposed method generalizes to forecasting tipping points occurring later in time and across different regimes than seen during training (e.g., different Reynolds numbers for the airfoil experiment), although the robustness depends on the generalizability of the models used to learn the nonstationary dynamics (Section 4.3).

## Supplementary Material

Appendix 01 (PDF)

## Data Availability

Code and training dataset have been deposited in Github (https://github.com/neuraloperator/tipping-pointforecast/) (70) and Zenodo (https://zenodo.org/records/18098807) (71). All other data are included in the manuscript and/or *SI Appendix*.
