# Supplementary material for: Tipping point forecasting in nonstationary dynamics on function spaces
Source: Proc Natl Acad Sci U S A. 2026 Oct 15;123(42):e2600331123. doi: 10.1073/pnas.2600331123 (PMC13684141; doi:10.1073/pnas.2600331123)
Supplement: Supplementary file 1 [file pnas.2600331123.sapp.pdf]

Supporting Information for

Tipping Point Forecasting in Non-Stationary Dynamics on Function Spaces

Miguel Liu-Schiaffini, Clare E. Singer, Nikola Kovachki, Sze Chai Leung, Hyunji Jane Bae, Kamyar Azizzadenesheli, and Anima Anandkumar

Anima Anandkumar
E-mail: anima@caltech.edu

This PDF file includes:

Supporting text
Figs. S1 to S21
Tables S1 to S6
Legends for Dataset S1 to S2
SI References

Other supporting materials for this manuscript include the following:

Datasets S1 to S2

Supporting Information Text

S1. Lorenz-63 experimental results

We consider tipping point forecasting in the non-stationary chaotic Lorenz-63 system (1), a simplified model for atmospheric dynamics. The non-stationary Lorenz-63 system is given by

$$\dot{u}_x = \sigma(u_y - u_x), \qquad \dot{u}_y = u_x(\rho(t) - u_z) - u_y, \qquad \dot{u}_z = u_x u_y - \beta u_z, \tag{S1}$$

where the state space is $u = (u_x, u_y, u_z)$, and the parameters of the system are $\sigma, \rho(t), \beta$, where ρ depends on time as in (2).

We find that RNO substantially outperforms MNO and RNN in learning the evolution of the non-stationary system. Furthermore, we use the Lorenz-63 system as a case study to justify our tipping point forecasting framework. Figure 1b shows that RNO is capable of accurately forecasting the tipping point 64 seconds ($T = 200 \cdot \Delta T$) ahead, even though the size of each input/output interval is $\Delta T = 0.32$ seconds.

We also demonstrate that our method is capable of forecasting tipping points far in advance *using only approximate knowledge of the underlying system*. In particular, we perturb the parameters σ, ρ, β by some fixed quantity η and show that the error in the tipping point forecast for the Lorenz-63 system is still less than 1 second, even when predicting 64 seconds in advance.

In this paper, we follow the setting of (2) where $\sigma = 10$, $\beta = 8/3$, and ρ depends on time via the parameterization $\rho(t) = \rho_0 + \rho_1 \exp(t/\gamma)$, where $\rho_0 = 154$, $\rho_1 = 8$, and $\gamma = 100$.

A tipping point for this system occurs at approximately $\rho^* = 166$ (3), which corresponds approximately to time $t^* = 40$ under the parameterization above. This tipping point is induced by an intermittency route to chaos (3) as the dynamics transition from periodic $t < t^*$ to chaotic $t > t^*$. The tipping point can be classified as an instance of bifurcation-induced tipping (4) at a saddle-node bifurcation (2). Details on data generation and experimental setup can be found in Appendix S8.

S1.A. Learning non-stationary dynamics. Numerical results from our experiments are shown in Table S1. We observe that RNO outperforms MNO in relative L^2 error by *at least an order of magnitude* for every n-step prediction setting up to and including 32-step prediction. We observe that even when composed with itself 32 times, RNO is capable of maintaining relative L^2 error under 0.05. Further, we find that despite training MNO with a multi-step procedure up to $M = 12$ steps, this still does not prevent MNO error from steadily increasing as the number of steps to compose increases. While RNN is somewhat stable in its error when composed multiple times, RNO vastly outperforms it in L^2 error. We attribute the performance of RNO to its status as an architectural generalization of MNO to systems with memory. Further, RNO's discretization invariance and adaptability to function spaces makes it an improvement over fixed-resolution RNNs.

S1.B. Tipping point forecasting: fully-known physics. As described in Section 1, our conformal prediction approach for tipping point prediction relies on a model trained in pre-tipping dynamics. We then monitor variations in the physics constraint loss of model forecasts to predict tipping points in the future. Recall that the physics constraint loss is only a function of the model predictions $\widehat{u}$ and the time t. We define the physics constraint loss to be

$$\ell_P(\widehat{u}, t) = \left\| \dot{\widehat{u}}(t) - \widehat{\dot{u}}(t) \right\|_2, \tag{S2}$$

where $\dot{\widehat{u}}$ is the time-derivative* of the model's forecasted trajectory at time t and $\widehat{\dot{u}(t)}$ is the time derivative defined by the Lorenz-63 system (eq. S1) using the model's predicted state $\widehat{u}(t)$ at time t. That is,

$$\widehat{\dot{u}_x} = \sigma(\widehat{u}_y - \widehat{u}_x), \qquad \widehat{\dot{u}_y} = \widehat{u}_x(\rho(t) - \widehat{u}_z) - \widehat{u}_y, \qquad \widehat{\dot{u}_z} = \widehat{u}_x \widehat{u}_y - \beta \widehat{u}_z. \tag{S3}$$

Thus, ℓ_P is minimized when the time derivative of the model's predictions is equal to the expected derivative that all solutions to the Lorenz-63 system must satisfy (Eq. S1). In practice, ℓ_P is computed over an entire calibration set, which is unseen during training, and these samples are used to construct an empirical CDF of the physics loss.

In our experiments, we set $\Delta T = 0.32$ seconds. Here we study the difficult task of forecasting the tipping point 64 seconds ahead, corresponding to two hundred times the time scale of the model. For a fixed $\delta = 10^{-3}$, Figure S20a shows the effect of varying the critical loss threshold $l(\alpha)$ for a given α on RNO and MNO tipping point predictions.

From Figure S20a we make a key observation: the distribution of RNO's physics loss has both smaller mean and variability than that of MNO, as well as a shorter right tail. This is deduced from the observation that the first sudden spike in Figure S20a corresponds to the mode of the histogram of each model's physics losses over the calibration set. Furthermore, observe that the loss range between this spike and the time when the model achieves its delay in the forecasted tipping point of the smallest magnitude is much smaller for RNO than for MNO, implying the difference in the right-tail lengths of the two models. In particular, this analysis of the distribution of the physics loss for each model reinforces the results in Section S1.A that RNO is more successful than MNO in learning the underlying non-stationary dynamical system. Figures 1b and S1 show RNO achieving near-zero error in forecasting the tipping point 64 seconds ahead.

* Note that $\widehat{\dot{u}(t)}$ can in principle be computed to arbitrary precision due to the discretization invariance of RNO in time. In practice, we find that approximating $\widehat{\dot{u}(t)}$ using finite difference methods is sufficient.

S1.C. Tipping point forecasting: approximate physics. In this section, we investigate our method's performance in tipping point forecasting using *approximate physics constraints.* For instance, such a setting may occur when certain coefficients in a PDE or ODE are known only approximately. In this section, we consider this example of approximate physics for the nonstationary Lorenz-63 system, where we evaluate our proposed framework's capability of forecasting tipping points under different perturbations of the ODE parameters. Specifically, we again define the physics loss as in Eq. S2, but in this case, we redefine $\widehat{u}(t)$ by replacing $\sigma, \rho(t), \beta$ with $\tilde{\sigma} = \sigma + \eta, \tilde{\rho}(t) = \rho(t) + \eta, \tilde{\beta} = \beta + \eta$, respectively, where η is some fixed perturbation.

We use the same experimental setup and as in Section S1.B. As shown in Fig. S3, our proposed framework is robust to perturbations in the physical constraints and is able to forecast tipping points with very low error far in advance. This is particularly useful for real-world scenarios in which only approximate physical laws are known for a given system.

In particular, we observe that with even approximate physical knowledge of the underlying system, the notion of using the physics loss to verify the correctness of the model's predictions does not break down. This can be observed in Figures S3c and S3d, where a "bump" in the approximate physics loss can be seen at the forecast time, signaling the shift in distribution of the underlying dynamics.

S2. Non-stationary Kuramoto–Sivashinsky experimental results

We demonstrate the effectiveness of RNO and our tipping point forecasting method in PDE systems by considering the 1-d non-stationary KS equation, which takes the form

$$\frac{\partial u}{\partial t} + u\frac{\partial u}{\partial x} + \frac{\partial^2 u}{\partial x^2} + \kappa(t)\frac{\partial^4 u}{\partial x^4} = 0 \qquad \text{[S4]}$$

where $\kappa(t)$ is some time-dependent parameter of the system, $u(x,t)$ is a function of space $x \in [0, 2\pi]$ and time $t \in (0, \infty)$, and the initial condition is $u(\cdot, 0) = u_0$. Following (2), we parameterize $\kappa(t) = \kappa_0 + \kappa_1 \exp(t/\gamma)$, where $\kappa_0 = 0.0753$, $\kappa_1 = 0.0034$, and $\gamma = 75.3$. This system exhibits a tipping point near $\kappa^* = 0.08$ from periodic dynamics to chaotic dynamics. The tipping point can be classified as an instance of bifurcation-induced tipping (4) at a saddle-node bifurcation (2).

Analogously to the Lorenz-63 case (Eq. S2), we define the KS physics loss to be

$$\ell_P(\widehat{u}, t) = \left\| \frac{\partial \widehat{u}}{\partial t} + \widehat{u}\frac{\partial \widehat{u}}{\partial x} + \frac{\partial^2 \widehat{u}}{\partial x^2} + \kappa(t)\frac{\partial^4 \widehat{u}}{\partial x^4} \right\|_2 . \qquad \text{[S5]}$$

In our experiments, we consider the setting of forecasting tipping points far ahead in time, at several orders of magnitude longer time scales than the model is trained on. We compare the performance of RNO in tipping point forecasting with the performance of MNO, for varying values of α, the false-positive rate of our method. Note that as shown in Table 2, the relative L^2 error of RNN on forecasting the evolution of the KS equation is an order of magnitude larger than that of RNO and MNO.

For the KS equation, we set $\Delta T = 1.6$ seconds and seek to forecast the tipping point $T = 16$ seconds ahead. As shown in Figure S4, we observe that for all values of α shown, RNO vastly outperforms MNO in the proximity of tipping point prediction to the true tipping point of the system. At short forecasting intervals (e.g., $T = \Delta T$ or $T = 2 \cdot \Delta T$), RNO and MNO both accurately capture the tipping point, but at long time-scales only RNO retains good performance. This can likely be attributed to the lower performance of MNO in learning the nonstationary system dynamics (see Table 2), which causes MNO to produce fluctuating physics losses even in the pre-tipping regime, whereas RNO has a low, stable physics loss during pre-tipping.

Figure S20b compares the critical physics loss threshold $l(\alpha)$ for RNO and MNO when forecasting the KS tipping point 16 seconds ahead. For lower physics loss thresholds, RNO forecasts the tipping point with much better performance than MNO. As the critical physics loss threshold is increased, the performance of RNO improves slowly, suggesting that the distribution of RNO's physics loss has little weight on the right tail. In contrast, MNO exhibits the opposite behavior, suggesting that it is less reliable than RNO in learning the non-stationary dynamics. Crucially, we note that comparisons on the accuracy of tipping point predictions between two methods must be made at a fixed false-positive rate α (e.g., see Figure S4). In Figures S20b and S20a, we only seek to compare the distributions of physics loss and each model's respective ability to learn the underlying dynamics.

S3. Background on simplified cloud cover model

The model developed in (5, 6) is an extension of a traditional bulk boundary layer model (7), which represents the state of the atmospheric boundary layer and cloud by five coupled ordinary differential equations:

$$\frac{dz_i}{dt} = w_e(\Delta R, s) - Dz_i + w_{\text{vent}}(\text{CF}) \qquad \text{[S6a]}$$

$$\frac{ds}{dt} = \frac{1}{z_i}\left[V \cdot (s_0(\text{SST}) - s) + w_e(\Delta R, s) \cdot (s_+ - s) - \Delta R(\text{CO}_2, q_{t,+})\right] + s_{\text{exp}} \qquad \text{[S6b]}$$

$$\frac{dq_t}{dt} = \frac{1}{z_i}\left[V \cdot (q_{t,0}(\text{SST}) - q_t) + w_e(\Delta R, s) \cdot (q_{t,+} - q_t)\right] + q_{t,\text{exp}}(\text{SST}) \qquad \text{[S6c]}$$

$$\frac{d\text{CF}}{dt} = \frac{f_{\text{CF}}(\mathscr{D}) - \text{CF}}{\tau_{\text{CF}}}. \qquad \text{[S6d]}$$

$$C\frac{d\text{SST}}{dt} = \text{SW}_{\text{net}}(\text{CF}) - \text{LW}_{\text{net}} - \text{LHF}(\text{SST}, s, q_t) - \text{SHF}(\text{SST}, s) - \text{OHU}. \qquad \text{[S6e]}$$

The model is formulated such that CO_2 is the only external parameter and all other processes are represented by physically motivated, empirical formulations, with their parameters based on data from satellite observations or high-resolution simulations.

The first three equations physically represent conservation of mass Eq. (S6a), energy Eq. (S6b), and water Eq. (S6c), and the final two are equations for the cloud fraction and the sea surface temperature. In Eq. (S6a), z_i is the depth of the boundary layer [m], w_e is the entrainment rate which is itself parameterized as a function of the radiative cooling and the inversion strength, D is the subsidence rate [s^{-1}], w_{vent} is an additional additive entrainment term used to parameterize ventilation and mixing from overshooting cumulus convective thermals. In Eq. (S6b) and Eq. (S6c), s is the liquid water static energy [J kg^{-1}] and q_t is the total water specific humidity [kg kg^{-1}]. V is the surface wind speed [m s^{-1}], ΔR is the cloud-top radiative cooling per unit density [W m kg^{-1}], which is a function of CO_2 and H_2O, and s_{exp} and $q_{t,\text{exp}}$ are export terms representing the effect of large-scale dynamics (synoptic eddies and Hadley circulation) transporting energy and moisture laterally out of the model domain into other regions.

The cloud fraction is modeled as a linear relaxation on timescale τ_{CF} to a state $f_{\text{CF}}(\mathscr{D})$ which depends on the degree of decoupling in the boundary layer:

$$f_{\text{CF}}(\mathscr{D}) = \text{CF}_{\max} - \frac{\text{CF}_{\max} - \text{CF}_{\min}}{1 + \frac{1}{9}\exp(-m(\mathscr{D} - \mathscr{D}_c))} \quad \text{[S7a]}$$

$$\mathscr{D} = \left(\frac{\text{LHF}}{\rho \Delta R}\right)\left(\frac{z_i - z_b}{z_i}\right). \quad \text{[S7b]}$$

This parameterization is inspired by theoretical and observational work from (8, 9) and parameters $\text{CF}_{\max}$, $\text{CF}_{\min}$, and m are fit to data from (10–12).

Equation Eq. (S6e) is the standard surface energy budget equation for SST. On the left-hand side, $C = \rho_w c_w H_w$ is a heat capacity per unit area, where ρ_w and c_w are the density and specific heat capacity of water and H_w is the depth of the slab ocean. The value of H_w is arbitrary (here 1 m): it affects the equilibration time, but not the equilibrium results, which is appropriate given that the forcing is much slower than the equilibration timescale (approx. 50 days). On the right-hand side are the source terms from shortwave and longwave radiation, latent and sensible heat fluxes, and ocean heat uptake. Ocean heat uptake is solved for implicitly such that SST = 290 K for CO_2 = 400 ppmv and assumed constant in time.

To generate the data for our experiments, we set the CO_2 forcing to follow

$$CO_2(t) = c_0(1 + r)^t + \sigma W(t), \quad \text{[S8]}$$

where t is the time (in years), $W(t)$ is a Wiener process, c_0 is the initial CO_2 concentration, r is the annual rate of CO_2 increase, and σ is the scaling parameter for $W(t)$. We use $c_0 = 400$ ppm, $r = 0.1$, and $\sigma = 2.5$.

S4. Details on dataset for airfoil experiments

The flow simulation uses a finite-volume, unstructured-mesh large-eddy simulation (LES) solver (13). The spatially-filtered incompressible Navier-Stokes equations are solved with second-order accuracy employing cell-based, low-dissipative, and energy-conservative spatial discretization and a fully-implicit, fractional-step time-advancement method with the Crank–Nicholson scheme. The Poisson equation for pressure is solved using the bi-conjugate gradient stabilized method (14). The subgrid-scale stress is modeled using the dynamic Smagorinsky model (15, 16).

A NACA 0012 airfoil with chord length C is placed horizontally at the origin of the coordinate system as shown in Figure S5. Boundary conditions for the airfoil surface are defined as solid no-slip walls. The simulation domain is periodic in the spanwise direction. Uniform inflow of U_∞ is set to come in through the left and bottom surfaces of the domain. Additionally, convective outflow boundary conditions are set for both the top and right surfaces of the domain.

The computational meshes used in the simulations consist of approximately 200,000 cells. As illustrated in Figure S5, the airfoil is enclosed by a C-type mesh (17), which features approximately 250 cells along the airfoil's circumference. The boundary layer on the airfoil is captured by a mesh with $\Delta y_1/C \approx 0.004$, where Δy_1 is the height of the first wall-normal cell. Since our experiments focus on two-dimensional flow fields, the mesh is extruded in the spanwise direction using only three uniform cells, covering a total non-dimensional spanwise length of $L_z/C = 0.002$.

The resulting velocity fields sampled on a coarse grid, which spans from -2 to 4 in the x-direction and from -2 to 2 in the y-direction, with a uniform grid spacing of 0.01. For computational efficiency, we train and evaluate our models on a subset of this physical domain, spanning from 0.05 to 2.5 in the x-direction and from -1 to 1.32 in the y-direction. The lift force is obtained by numerically integrating the surface pressure and shear stress components normal to the freestream. The lift coefficient is then calculated using the lift force by

$$C_L = \frac{F_L}{\frac{1}{2}\rho U_\infty^2 L_z C}, \quad \text{[S9]}$$

where F_L is the lift force and ρ is the fluid density.

Ten unique trajectories with different initial conditions are simulated for Reynolds numbers of 1000 and 5000. Each trajectory is generated by continuously increasing the inflow angle of attack (AoA) at a constant rate of 0.33 degrees per time unit (tU_∞/C). The wake transition and stall tipping points are manually identified by examining the time history of the airfoil lift coefficients.

S5. Additional results on the cloud cover system

S5.A. Numerical results in learning non-stationary dynamics. Numerical results from our experiments are shown in Table S2. We observe that RNO outperforms MNO and RNN in relative L^2 error for every n-step prediction presented. We find that as the size of the forecasting interval ΔT (not to be confused with the length of the forecasting window, which is $n \cdot \Delta T$) increases, the performance of RNN worsens substantially. We attribute this to the resolution-dependence of RNN; as the dimensionality of the input (in this case $5 \cdot \Delta T$, since the cloud cover system is 5-dimensional) increases, the size of the RNN model must be increased accordingly in order to adequately capture the dynamics of the system. For RNO and MNO, the size of the models need not be increased substantially since the input is interpreted as a function over a larger domain, and the inductive biases of these models allows them to outperform fixed-resolution models such as RNNs.

S5.B. Tipping point forecasting using full ODE constraint. In this section, we demonstrate that our proposed method is capable of identifying the tipping point with low error when using the full ODE equation as the physics constraint. Figure S6 shows that our method is capable of forecasting the tipping point far in advance, similarly to Figure 5.

Similarly to the nonstationary KS setting, we also compare the performance of RNO in tipping point forecasting with MNO and RNN for varying values of false-positive rate α for the simplified cloud cover system (Figures S7 and S8). We observe that RNO overall outperforms RNN and MNO in tipping point forecasting for a variety of false-positive rates α. Furthermore, RNO is able to achieve nearly no increase in error as α increases, for reasonable α.

S6. Additional results on the airfoil tipping points

As mentioned in Section C, our airfoil experiments involves the three settings: (1) a Reynolds number (Re) of 1000, where only the static stall occurs, (2) the wake transition in a flow with Reynolds number 5000, and (3) stall in a flow with Reynolds number 5000. Each model learns the evolution operator mapping the initial state forward in time by $\Delta T = 0.1\ tU_\infty/C$.

Although the main goal of the airfoil experiments is to demonstrate the effectiveness of our proposed tipping points forecasting method, we present the L^2 relative test error for these experiments for completeness. The results for RNN, MNO, and RNO on the pre-tipping Re 1000 stall, Re 5000 wake, and Re 5000 stall settings can be found in Tables S3, S4, and S5, respectively. We present results for long-time rollouts for each model, going up to 25 steps for Re 1000 stall and Re 5000 wake and up to 8 steps for the harder Re 5000 stall setting.

For the Re 1000 stall and Re 5000 wake settings, we observe that the performance of each model is very similar, even up to autoregressively rolling out for 25 steps. This is likely attributable to the simpler dynamics of pre-tipping Re 1000 stall and Re 5000 wake, where the dynamics are smoother. In contrast, the pre-tipping L^2 errors in the Re 5000 stall setting are more interesting because the models must successfully forecast beyond the wake transition. We observe that RNN is unable to learn these dynamics and that the neural operator methods vastly outperform the finite-dimensional RNN. Interestingly, MNO outperforms RNO for the portion of the rollout beyond the training regime, whereas RNO outperforms MNO within the training regime of 3 autoregressive steps. However, RNO in general outperforms both MNO and RNO in forecasting the tipping points.

The airfoil tipping points in this paper are forecasted at a temporal horizon of $T = 1.0\ tU_\infty/C$ in advance. The divergence computation in the physics loss $\ell_P(u) = \|\nabla \cdot u\|_2$ is implemented using finite differences. Figure S9 shows a comparison of RNO, MNO, and RNN on the forecasting the Re 1000 stall transition. From the figure, we see that RNO produces the most stable physics loss pre-tipping, and it is thus the most accurate at forecasting the tipping point for the various choices of α.

We observe a similar trend in Figure S10 and Figure S11, where RNO performs at least as well as MNO and RNN in forecasting each tipping point. In this Re 5000 setting, we can more easily observe the benefits of neural operators: compared to RNO and MNO, the RNN physics loss is very noisy and oscillatory, particularly around the stall point. In contrast, both RNO and MNO exhibit a slight increase in physics loss around the wake tipping point and a larger increase around the stall tipping point. This behavior is seen in both cases when we train the models on pre-wake and pre-stall dynamics.

Finally, as shown in Section C, we explore the generalization ability of our methods beyond the regime of the training dynamics. Figure S12 shows that RNO, MNO, and RNN trained on pre-wake data can be used to accurately forecast the stall tipping point at inference time. Figure S13 shows the results of RNO trained on Re 1000 pre-stall dynamics can be used directly to forecast the Re 5000 wake tipping point, and Figure S14 shows the analogous result for the Re 5000 stall tipping point. In the latter two cases, we find that MNO and RNN lag behind RNO's generalization ability. All of the these trends hold true across a variety of α.

S7. Robustness to rate of parameter change and non-tipping scenarios

In this paper, we demonstrate the applicability of our method for forecasting tipping points across a variety of different systems, such as non-stationary Lorenz, non-stationary Kuramoto-Sivashinsky, a cloud cover dynamics model, and stall and wake transitions in a flow over an airfoil. For each of these experiments, we fix a rate of change of a critical system parameter to induce non-stationarity. In this section, we show that our method is robust to variations in this rate of change.

We select the more difficult airfoil setting for these experiments. We show that RNN, MNO, and RNO are all capable of forecasting stall transitions in the airfoil setting under varying rates of change. In particular, we train and evaluate models under the same setup as explained in Appendix S4 except with two different angles of attack: 1.32 and 5.0 degrees per time unit (which we shorten to 1.32 AoA and 5.0 AoA, respectively). The dynamics are qualitatively distinct from the 0.33-degrees case:

in the former, we predict the static stall transition and in the latter we predict a dynamic stall. See Figure S15 for more details. Comparisons between RNO, MNO, and RNN can be found in Figure S16 and Figure S17. In the 1.32 AoA case, we see that all three models are capable of forecasting the tipping point with high accuracy, whereas in the faster dynamic stall case (5.0 AoA setting), all three models tend to predict after the true tipping point for the α values studied. We observe that for larger values of α, the model can accurately forecast the tipping point, but for consistency with previous results and to maintain fairness in evaluation, we keep the three smallest values of α (rounded to two decimal places) permitted by the size of our calibration set.

We also train and evaluate our models on a non-stationary setting with no tipping point. In this setting, we keep the same 0.33 pitch rate as the airfoil experiments in the main paper, up to an angle of attack of 10 degrees, where we reverse the pitch rate until the angle of attack returns to zero. The results are shown in Figure S18. We compare across three false positive rates, where the first is the minimal possible (according to the calibration set size) α. For all three models, we see that the variance of the physics loss is substantially smaller than for the tipping cases. Further, we see that RNO and MNO can successfully avoid predicting a tipping point at the smallest α.

We note that by construction, it is difficult to avoid predicting a tipping point for any significance level α larger than the minimum allowable value when the evaluation trajectory is sufficiently long. In this case, the smallest admissible α (determined by the calibration set size) corresponds roughly to declaring a tipping point only when the observed physics error exceeds the maximum calibration error. As the calibration set grows, this threshold becomes increasingly effective at suppressing false positives.

Although α has a clear statistical interpretation as the significance level governing the conformal prediction procedure, its practical operating point inevitably depends on the quality of the learned dynamics model (e.g., RNO, MNO, RNN, etc.) and the application-specific tradeoff between early detection and false positives. When representative trajectories with known tipping events are available, practitioners may therefore select α empirically to achieve the desired operating characteristics prior to deployment.

S8. Details on numerical experiments

In our RNO experiments, we divide inference into a warm-up phase and a prediction phase. This choice is motivated by the empirical observation that predictions $\widehat{u}_1, \widehat{u}_2, \ldots$ for small steps τ tend to be poor because the hidden function $h_0(x,t)$ does not contain sufficient information to properly learn complex nonstationary dynamics (which inherently require history). The effect of the warm-up phase is to allow the RNO to construct a useful hidden representation h_τ before its predictions $\widehat{u}_\tau$ are used to construct long-time predicted trajectories. We use this procedure both during training and test times.

Furthermore, MNO has achieved state-of-the-art accuracy in forecasting the evolution of stationary dynamical systems by using a neural operator (Section 1) to learn the Markovian solution operator of such systems. As proposed in (18), MNO takes as input the solution at a given time, $u(x,t)$, and outputs $u(x,t+h)$. While the Markovian solution operator holds for some stationary systems (18), non-stationary systems must be handled with more care. As such, we instead compare RNO against a variant of MNO that maps $u(x,[t,t+\Delta T]) \mapsto u(x,[t+\Delta T, t+2\Delta T])$. This variant of MNO equips the model with some temporal memory.

We perform hyperparameter tuning (e.g., for model size, learning rates, etc.) for all architectures used in our experiments. We report our final hyperparameter values for each experiment below.

S8.A. RNN baseline. We use a gated recurrent unit (GRU) (19) architecture for the RNN baseline for the non-stationary Lorenz-63, KS equation, and simplified cloud cover model experiments. We use shallow neural networks to map inputs to a hidden representation that is propagated between time intervals with the GRU, and the final GRU hidden-state is mapped to the output space with another shallow neural network. We observe optimal performance for the RNN when we adopt the same inference process as with RNO, with a warm-up phase and a prediction phase. We observe that the number of time intervals for the warm-up phase needed to achieve acceptable results tends to be higher for RNN than for RNO across our experiments. For instance, we warm up with 10 intervals for RNN and 5 for RNO, in both the Lorenz-63 and KS settings. In the cloud cover experiments, we warm up the RNN and RNO both with 5 time intervals.

In Table S6, we note the effectiveness of multi-step fine-tuning in learning the evolution of longer trajectories in non-stationary systems (compare RNN-8 and RNN-1). However, if multi-step fine-tuning is taken too far (i.e., M is large), we empirically observe convergence to suboptimal local minima (e.g., RNN-12).

S8.B. Non-stationary Lorenz-63 system. In our experiments, we generate 15 trajectories using a fourth-order Runge-Kutta method with an integration step of 0.001. Ten of these trajectories are used for training and the remaining five are used for calibration and testing. Each trajectory is on the range $t \in [-600, 200]$. The initial condition for each trajectory is initialized randomly, and the solution is integrated for 50 integrator seconds and then discarded to allow the system to reach the periodic dynamics. For training and testing, the data is temporally subsampled into a temporal discretization of 0.05 integrator seconds.

Both the RNO and MNO used for experiments in Section 1 were trained with a width of 48 and 24 Fourier modes. See (20) for a description of the hyperparmaters of Fourier layers. We use $L = 3$ layers in the RNO and 4 layers for MNO. We implement the baseline RNN as described in Section S8.A, with 3 layers and a 248-dimensional hidden state. We set the length of each input/output time interval to $\Delta T = 0.32$ seconds. We also fix the number of warm-up samples $N_\tau = 5$ for RNO experiments. In our experiments, we set $M = 12$ and $\lambda_n = 1$ for all n, for the corresponding terms in our data loss (Eq. 12). All models were optimized using Adam (21) with an initial learning rate of 10^{-4} and a batch size of 64. We use a step learning

rate scheduler that halves the learning rate every 4000 weight updates. We train RNO and MNO for 5 epochs, and we train RNN for 15 epochs. We pre-train MNO for 50 epochs on one-step prediction.

S8.C. Non-stationary KS equation. In our experiments, we generate 200 trajectories of the evolution of the non-stationary KS equation using a time-stepping scheme in Fourier space with a solver time-step of 0.001. 160 of these trajectories were used for training, 30 of them were used for calibration, and 10 trajectories were used for testing. Each trajectory is defined on the range $t \in [0, 100]$. The initial condition is initialized randomly, and the solution is integrated for 100 integrator seconds and then discarded to allow the system to reach periodic dynamics. For training and testing, the data is temporally subsampled into a temporal discretization of 0.1 integrator seconds. Our models map the previous time interval of length ΔT to the next time interval of length ΔT. In our model, we use $\Delta T = 1.6$ seconds.

Both the RNO and MNO used in our experiments used 20 Fourier modes across both the spatial and temporal domain. The RNO used has a width of 28, and MNO used has a width of 32. We use $L = 3$ layers in the RNO and 4 layers for the MNO. We implement the baseline RNN with 3 layers and a 512-dimensional hidden state. We fix the number of warm-up samples $N_\tau = 5$ for RNO experiments. In our experiments, we set $M = 5$ and $\lambda_n = 1$ for all n, for the corresponding terms in our data loss (Eq. 12). All models were optimized using Adam (21) with a batch size of 32. RNO was trained for 25 epochs at an initial learning rate of 10^{-3}, while halving the learning rate every 2000 weight updates. MNO was pre-trained on one-step prediction for 100 epochs at a learning rate of 0.005, halving the learning rate every 200 weight updates. MNO was then fine-tuned using the loss in Eq. 12 at a learning rate of 10^{-5}, halving the learning rate every 250 weight updates. RNN was pre-trained on one-step prediction for 40 epochs at a learning rate of 0.001, halving the learning rate every 500 weight updates. MNO was then fine-tuned using the loss in Eq. 12 at a learning rate of 10^{-5}, halving the learning rate every 500 weight updates.

Figure S19 depicts the convergence in test L^2 error of RNO trained on the KS equation. We obseve that RNO converges to a test L^2 error close to zero for all steps. Note that at times, training is slightly unstable for 1-step prediction, and this error propagates to larger degrees for 2-step, 3-step, etc., predictions. Empirically, we do not observe that this behavior prevents RNO from adequately learning the multi-step dynamics. In fact, observe that after minor increases in 1-step error in Figure S19, when the 1-step error decreases, the multi-step error decreases accordingly.

S8.D. Cloud cover equations. We generate 150 trajectories of the evolution of the non-stationary cloud cover equations (5, 6) described in Appendix S3 using a 5th order Runge-Kutta Rosenbrock method. We use a integration step of 1 day and solve the system for 20 years for each trajectory. We train our RNO model on 45 trajectories, use 100 for calibration, and 5 for testing. In our models, we set the forecast time length to be $\Delta T = 0.36$ years.

The RNO used in our experiments used 64 Fourier modes across the temporal domain and used a width of 128. We use $L = 5$ layers and multi-step fine-tuning up to $M = 10$ steps. The MNO model used 4 layers, 32 Fourier modes, and a width of 96. We implement the baseline RNN with 3 layers and a 1024-dimensional hidden state. We fix the number of warm-up samples $N_\tau = 5$ for RNO experiments. In our experiments, we set $M = 10$ and $\lambda_n = 1$ for all n, for the corresponding terms in our data loss (Eq. 12). All models were optimized using Adam (21) with a batch size of 64. RNO was trained for 25 epochs at an initial learning rate of 10^{-3}, while halving the learning rate every 300 weight updates. Both MNO and RNN were also directly trained using the loss in Eq. 12 at a learning rate of 10^{-3}, halving the learning rate every 500 weight updates.

S8.E. Airfoil experiments. The details regarding data generation and problem setup can be found in Section S4. The RNO used in our experiments used 26 Fourier modes aong each spatial dimension and used a width of 28. We use multi-step fine-tuning up to $M = 3$ steps. For the Re 1000 experiments, we use $L = 3$ layers and $L = 4$ layers for Re 5000. The MNO model used 4 layers, 26 Fourier modes along each spatial dimension, and a width of 28. We implement the baseline RNN with 3 layers and a 64-dimensional hidden state. We fix the number of warm-up samples $N_\tau = 4$ for RNO experiments. In our experiments, we set $M = 3$ and $\lambda_n = 1$ for all n, for the corresponding terms in our data loss (Eq. 12). All models were optimized using Adam (21) with a batch size of 5. RNO was trained for 50 epochs at an initial learning rate of 10^{-3}, while halving the learning rate every 10 epochs. Both MNO and RNN were also trained for the same number of epochs and schedule. However, MNO used an initial learning rate of $5 \cdot 10^{-4}$, and RNN used a learning rate of 10^{-4}.

S9. Comparisons to prior works

S9.A. Comparison to prior machine learning methods. In recent years, there has been a several works using machine learning for tipping point forecasting and prediction (2, 22–32). For instance, (22) uses a convolutional long short-term memory (LSTM) model to predict specific types of bifurcations. (27) proposes a similar methodology of classifying critical transitions, smooth transitions, and no transitions. (29) proposes to use a 1-d CNN to predict the probabilities of rate-induced tipping events. However, such methods are not directly applicable to the large-scale spatiotemporal systems (i.e., on function spaces) that motivate our work and require access to post-tipping data. In another vein of research, reservoir computing (RC)-based methods have been used to learn the dynamics of 3d-Lorenz equation (2, 23–26, 30). However, RC approaches do not operate on function spaces and are thus not suitable for many large-scale spatiotemporal scientific computing problems.

More specifically, (2) makes the observation that when a tipping point happens, a well-trained machine learning model on the pre-tipping regime makes a significant error, using this signal as an indicator for tipping points. However, this method requires the use of post-tipping information to compare their model forecasts against. In our work, we extend this observation into our proposed tipping point forecasting method that does not require post-tipping data. Instead, we compare our forecast against the physics constraints or differential equations driving the underlying dynamics.

Among other reservoir computing works, (24) and (30) train data-driven reservoir models on pre-tipping dynamics, conditioning the model on certain values of a bifurcation-inducing external parameter. While such methods appear successful for simpler toy systems, for real-world systems (e.g., climate) there may be a variety of external parameters that may affect the dynamics of a non-stationary system, which may be difficult to estimate and identify. (32) addresses this by using a variational autoencoder to estimate latent driving factors from data directly, without requiring prior parameter knowledge. However, their method is based on RC and does not natively extend to function spaces or to large spatiotemporal systems at arbitrary resolutions. (25) also use reservoir computing but require access to the full ground-truth system, which may be unknown in real-world systems. In contrast, our method can operate on partial or approximate physics knowledge of the underlying system. (26) also presents a method for tipping point prediction using reservoir computing, but this method suffers from the same scalability concerns of reservoir computing and also requires tipping points to be in the training set, both of which are addressed by our method.

In another realm of methods, (28) introduces an adversarial framework for tipping point prediction. However, this framework requires the querying of an oracle, which is extremely computationally expensive in large-scale spatiotemporal systems of interest. Another interesting direction is proposed in (31), which proposes a multi-step procedure of dimensionality reduction, learning a mesoscopic dynamical model, and identifying tipping points via numerical bifurcation analysis of this model. While this procedure proposes a unique direction compared to many prior works, it has yet to be shown whether its components can be scaled to the complex real-world systems that motivate our work.

S9.B. Comparisons with traditional early warning signals. To analyze the ability of traditional early warning signals (EWS) to predict tipping points beyond simple well-studied systems, we apply EWS to the cloud cover system (5, 6) in our experiments. In particular, prior works have found that for some systems, increases in variance and autocorrelation are associated with critical slowing down, a phenomenon of slow recovery from perturbations for some systems approaching bifurcation-induced tipping points (33, 34). As such, we compute the variance and lag-1 autocorrelation using the methods in (34) for each of the five variables in the cloud cover system (see Appendix S3 for background). The variance and autocorrelation for three of these variables is shown in Figure S21.

Despite autocorrelation and variance being established indicators of tipping phenomena in some systems, we observe that in this real-world system, autocorrelation never has a positive Kendall τ, and variance only has a positive Kendall τ in the cloud fraction setting. These results suggest that traditional EWS are not reliable as general indicators of tipping phenomena.

We follow the methodology described in (34), and we compute the variance and autocorrelation using the `ewstools` package (35). We smooth each univariate time-series using LOWESS smoothing with a span of 0.1 times the length of the trajectory. We compute lag-1 autocorrelation and variance in rolling windows of length 0.25 times the length of the trajectory.

S10. Technical discussion and analysis of methods

S10.A. Memory and inference times. The details for the best-performing RNO and MNO models in each of the experimental settings are provided in Appendix S8. In this section we discuss and compare the memory usage and the inference times of RNO and MNO. Timing benchmarks were performed on one NVIDIA P100 GPU with 16 GB of memory.

In general comparisons of RNO and MNO with the same number of layers, Fourier modes, and width, RNO tends to have a significantly larger memory footprint, since all of the gating operations are implemented with Fourier integral operators. However, in situations where memory may be scarce, weight-sharing or factorization methods can be used to vastly reduce memory footprint (36). Mixed-precision neural operators (37) can also be used to dramatically decrease memory usage and inference times. Apart from the additional Fourier layers for the gating operations, RNO inference has a warm-up period of N_τ steps to achieve a reasonable hidden state representation. This warm-up period scales with $O(N_\tau)$, since the computation is computed serially. Despite RNO's longer inference times compared to MNO, we find that these inference times are still much faster than the numerical solvers for a variety of applications, particularly for larger-scale spatiotemporal problems.

For the non-stationary Lorenz-63 setting, our RNO model and MNO model both used 24 Fourier modes and had a width of 48. The respective memory usages are 8.1 MB and 1.9 MB, respectively. The respective inference times to generate an entire trajectory is 30.8 seconds and 11.1 seconds, respectively (averaged over 10 instances). While RNO is slower than MNO, it is still significantly faster than the numerical solver, which takes around 90 seconds to generate each trajectory.

For the non-stationary KS equation, we compare RNO and MNO models with 20 Fourier modes across both time and space and a width of 28. The respective memory usages are about 90 MB and 7 MB, and the respective inference times to generate a full trajectory are about 1.3 seconds and 0.3 seconds (both averaged over 100 instances). These are both significantly faster than the numerical solver, which takes about 19 seconds to simulate one trajectory.

For the simplified cloud cover experiments, the best-performing RNO model used 64 Fourier modes and had a width of 128, whereas the best-performing MNO model used 32 Fourier modes and had a width of 96. The corresponding memory usages are 254 MB and 10 MB, respectively. However, note that while these models are the best-performing for their architecture, they do not have the same hyperparameters. MNO with 64 modes and width 128 has a memory footprint of 34 MB. The inference time to generate an entire trajectory for RNO is 1.068 seconds, and for MNO the inference time is 0.223 seconds, averaged over 100 instances. Note that these are both orders of magnitude faster than the numerical solver, which takes about 100 seconds to generate an entire trajectory.

S10.B. On the choice of hyperparameters for RNO. The primary hyperparameters of our proposed RNO model is L, the number of RNO layers, the number of Fourier modes across each dimension of the input, the width (i.e., co-dimension) of each RNO layer, N_τ, the number of warm-up intervals, and M, the number of auto-regressive steps used during training.

In general, RNO's L and width is analogous to the depth and width of standard neural networks, or the number of layers and dimensionality of the hidden state in RNN's. Increasing L allows for more non-linear and expressive mappings to be learned, and the number of parameters in the model increases linearly with L. In general, under our implementation we observe that increasing L beyond 5 or 10 layers does not confer additional benefits for the systems studied in this paper. We also observe that increasing the width of RNO rarely improves performance significantly, only marginally, if at all.

The number of Fourier modes necessary depends heavily on the underlying dynamics of the data. For instance, for highly turbulent and chaotic fluid flows, a large of number of modes is useful to effectively parameterize and capture high-frequency information. On the other hand, for laminar fluid flows, a large number of Fourier modes may not be necessary.

Adjusting the optimal number of auto-regressive steps M used during training is a trade-off between ease of training and performance (in L^2 error) on longer time horizons. For large values of M, optimization may be difficult and complicated training policies (e.g., progressive steps of pre-training for some $\tilde{M} < M$, then fine-tuning at M, etc.) may be necessary to improve performance. However, we find that using some $M > 1$ is crucial to achieving good long-term performance. This trade-off can be observed in Table S6. In general, the choice of M may also depend on the system and the goals for the learned surrogate model. For instance, for highly chaotic systems, large M are less likely to provide benefit over smaller M, since chaotic systems tend to quickly decorrelate from the past, making precise long-term predictions very challenging (18).

Finally, the choice of the number of warm-up intervals N_τ also depends on the degree of non-stationarity of the system. For stationary Markovian systems, no memory warm-up is needed, in principle, so $N_\tau = 0$. For highly non-stationary systems (especially those with many latent variables), increasing N_τ can lead to an improvement in model performance. Also, for chaotic systems that decorrelate from the past quickly, large values of N_τ are likely to not bring better performance. In general, we find that N_τ need not be very large; values between 3 and 10 appear to work well for most systems. We also observe that RNO performance appears to be quite robust to N_τ, and tuning this hyperparameter often does not bring significant changes in performance.

S10.C. Limitations and discussion of proposed methods. As with all data-driven methods, the performance of RNO depends on its training data. On one hand, data-driven approaches are flexible, unconstrained, and have been shown to learn complex dynamics purely from data (18, 38). On the other hand, purely data-driven approaches often lack theoretical guarantees; in the realm of scientific computing such useful guarantees may be adhering to physically-meaningful conservation laws, for instance.

Despite lacking many theoretical guarantees, our proposed conformal prediction method is capable of quantifying the distribution of model error with respect to the physics loss. That is, our method does not assume that the data-driven RNO (or other data-driven surrogate model) follows physical constraints perfectly in the pre-tipping regime. Using our rigorous proposed uncertainty quantification method, our tipping point prediction method is robust to model errors.

Since RNO is purely data-driven, it can, in principle, be applied to learning the dynamics of arbitrary dynamical systems. For stationary dynamical systems, it is possible for RNO to learn to ignore historical information and thus to simplify to MNO. Unfortunately, if data is sparse or of low-resolution, it may be difficult for RNO to generalize and truly learn the underlying evolution operator of the system. Providing a heuristic rule-of-thumb for the minimum resolution of the training data that RNO needs to adequately learn the dynamics is difficult; this largely depends on the dynamics of the system in question. In general, providing training data at which the underlying physics is not resolved introduces an ill-posed learning problem. On the question of minimum amounts of training data, we find empirically that simple dynamics can be learned with $O(1000)$ training pairs, whereas complex dynamics may need $O(10000)$ training pairs to adequately learn. This is a very coarse estimate derived from our experimental settings and may not generalize to arbitrary systems.

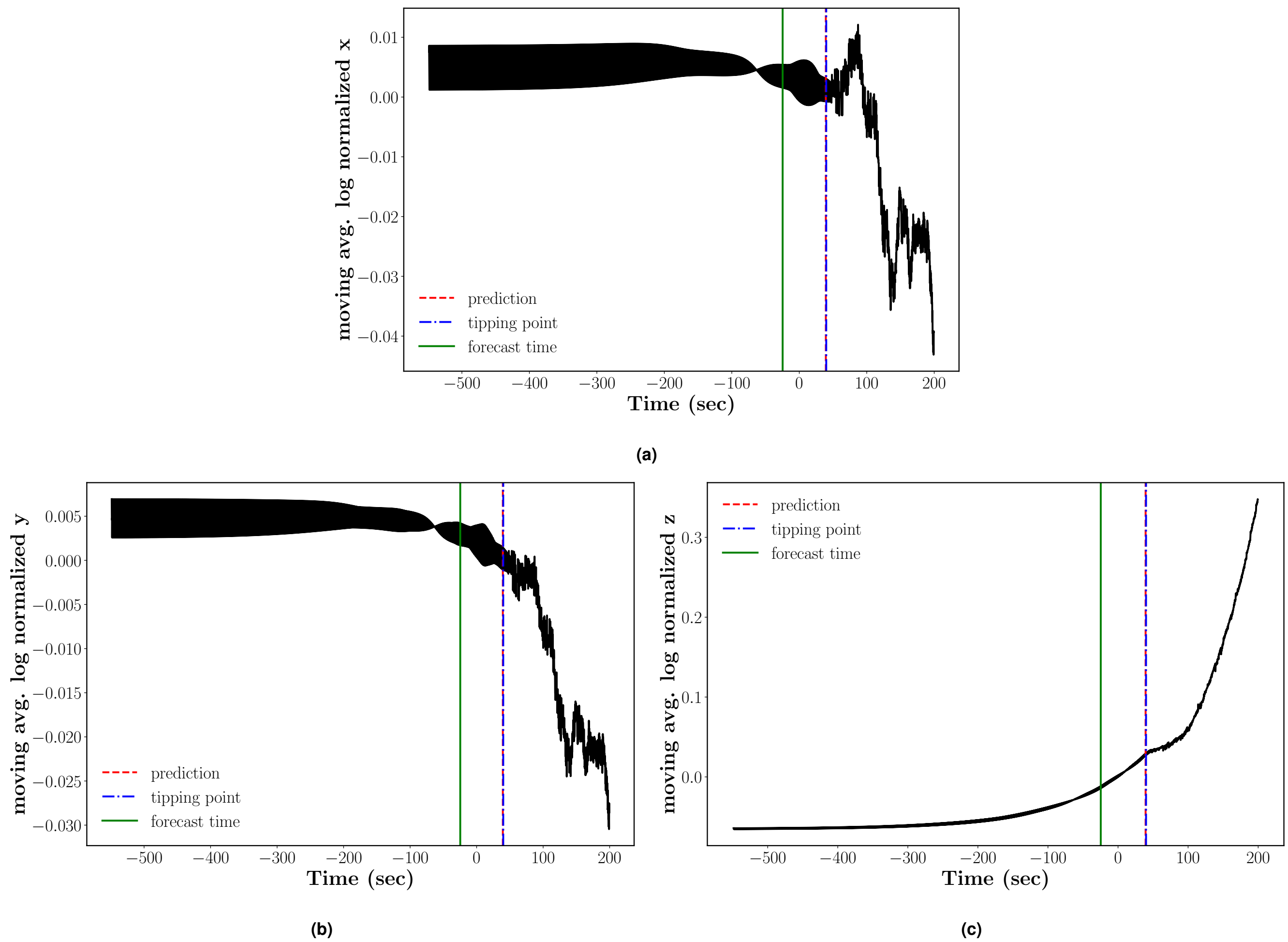

Fig. S1. Log normalized components of the non-stationary Lorenz-63 system plotted with a 50-second moving average. The predicted tipping point aligns nearly exactly with the true periodic-chaotic tipping point of the system, which can be seen as a qualitative change in dynamics of the system components. A false-positive rate of $\alpha = \varepsilon \approx 0.03$ is used, where ε is discussed in Section 1.

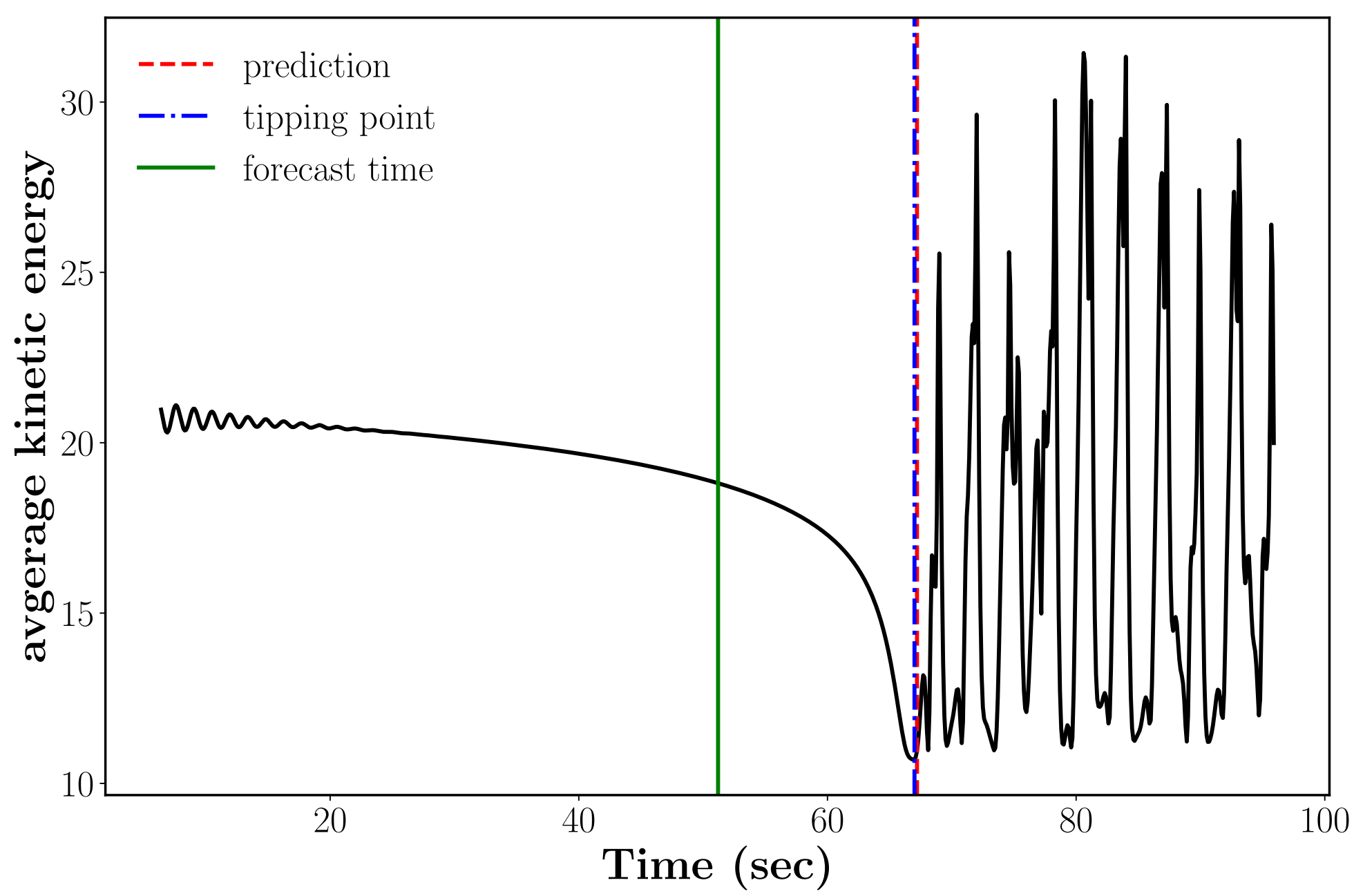

Fig. S2. Kinetic energy of the non-stationary KS system. The predicted tipping point aligns nearly exactly with the true tipping point of the system, which can be seen as a qualitative change in the kinetic energy of the system. The false-positive rate is $\alpha = 0.07$.

Model	1-step	2-step	4-step	8-step	16-step	32-step	64-step	128-step
RNO	**0.0053**	**0.0033**	**0.0027**	**0.0028**	**0.0068**	**0.0434**	**0.1549**	**0.2585**
MNO	0.0156	0.0242	0.0311	0.0579	0.1035	0.1638	0.2274	0.3114
RNN	0.0141	0.0139	0.0173	0.0150	0.0332	0.1096	0.2366	0.3434

Table S1. Relative L^2 errors on non-stationary Lorenz-63 for different n-step prediction settings. RNO outperforms MNO on every forecasting interval and maintains low error even for long intervals.

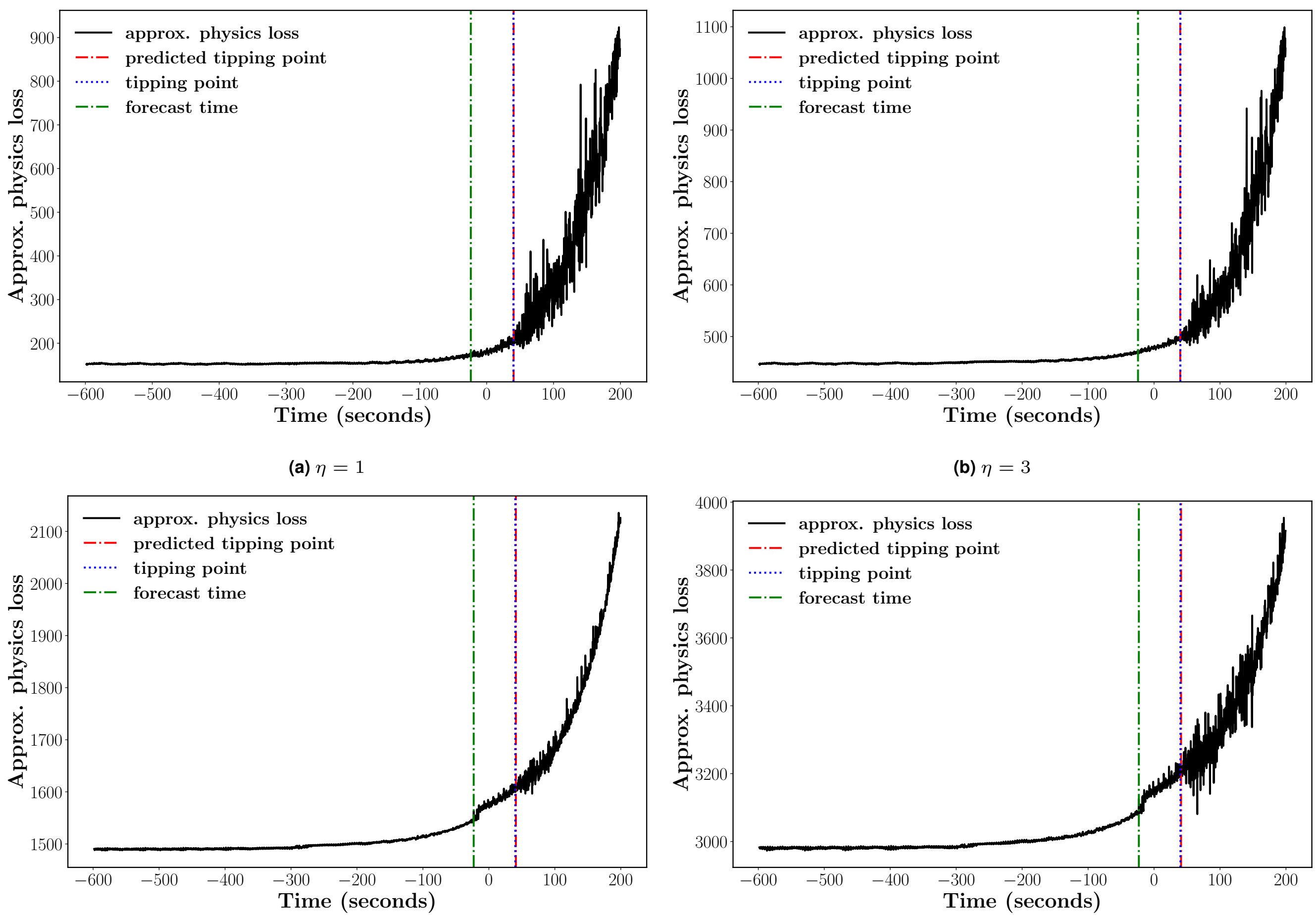

(a) $\eta = 1$

(b) $\eta = 3$

(c) $\eta = 10$

(d) $\eta = 20$

Fig. S3. Lorenz-63 tipping point forecasting performance 64 seconds ahead using approximate physical constraints, where η is the perturbation on the ODE parameters for the physical law. Our framework is capable of forecasting the tipping points with very low error even with large perturbations η. A false positive rate of $\alpha = \varepsilon \approx 0.03$ is used, where ε is discussed in Section 1.

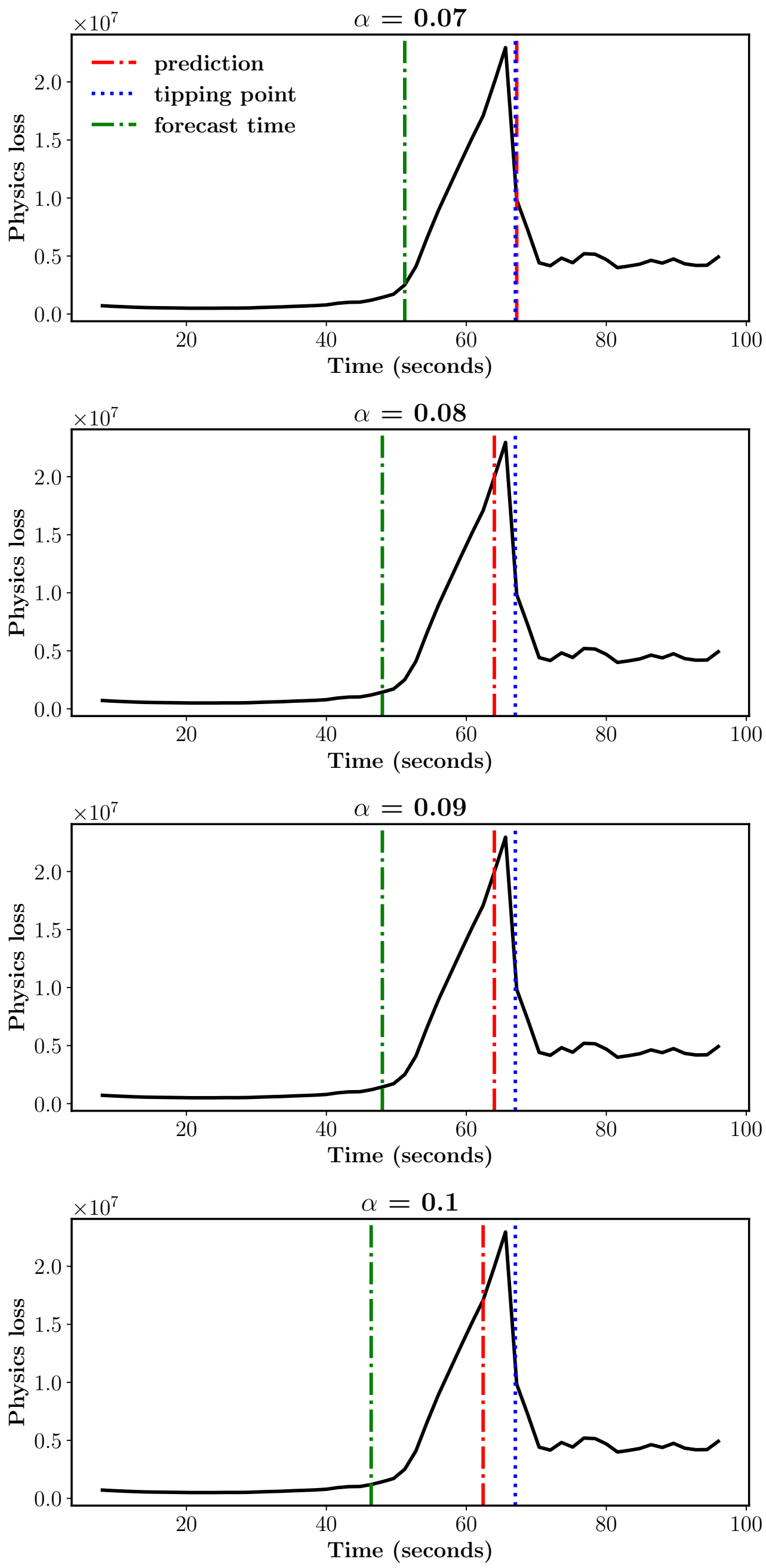

(a) RNO

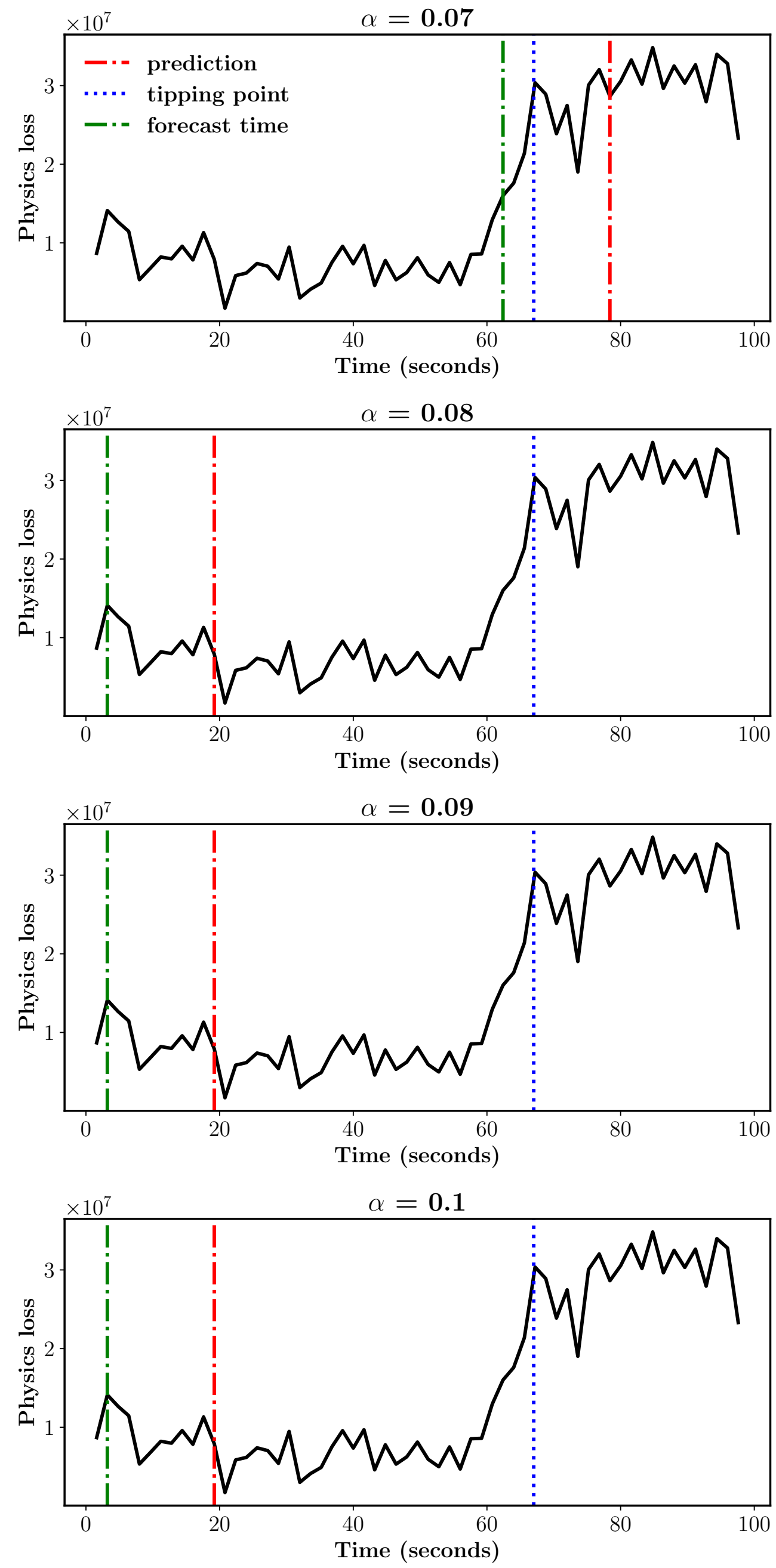

(b) MNO

Fig. S4. Comparison of tipping point forecasting performance between RNO and MNO for various false-positive rates α for the KS equation, forecasting 16 seconds ahead. For all presented α, RNO outperforms MNO in tipping point forecasting error.

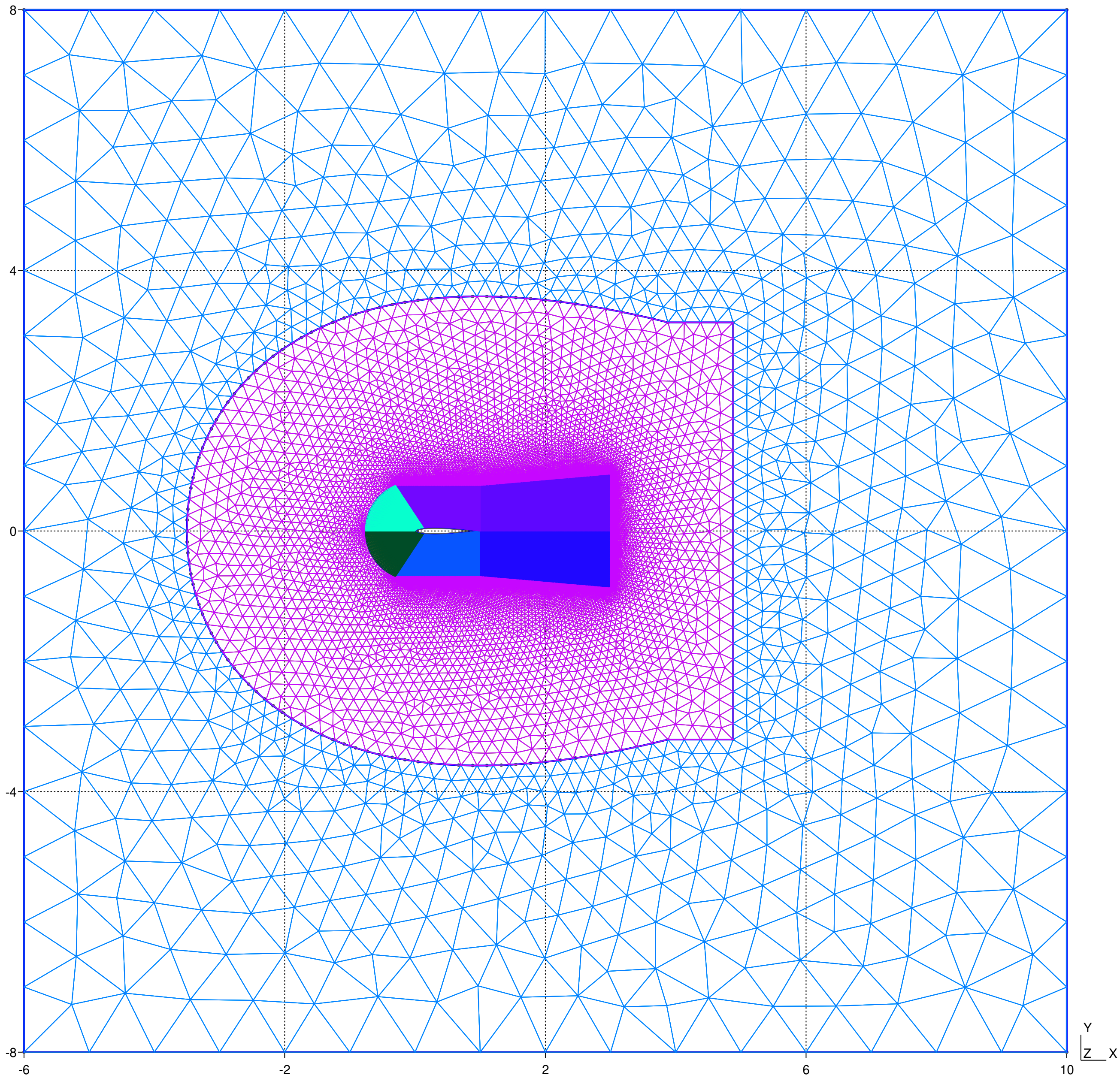

Fig. S5. Computational mesh of the airfoil used in the simulations.

Model	1-step	2-step	4-step	8-step	16-step
RNO	**0.0234**	**0.0326**	**0.0449**	**0.0614**	**0.0852**
MNO	0.0246	0.0342	0.0472	0.0647	0.0860
RNN	0.0285	0.0360	0.0481	0.0645	0.0868

Table S2. Relative L^2 errors on the cloud cover system for different n-step prediction settings using the experimental setup described in Appendix S8.D. RNO outperforms MNO and RNN on every forecasting interval.

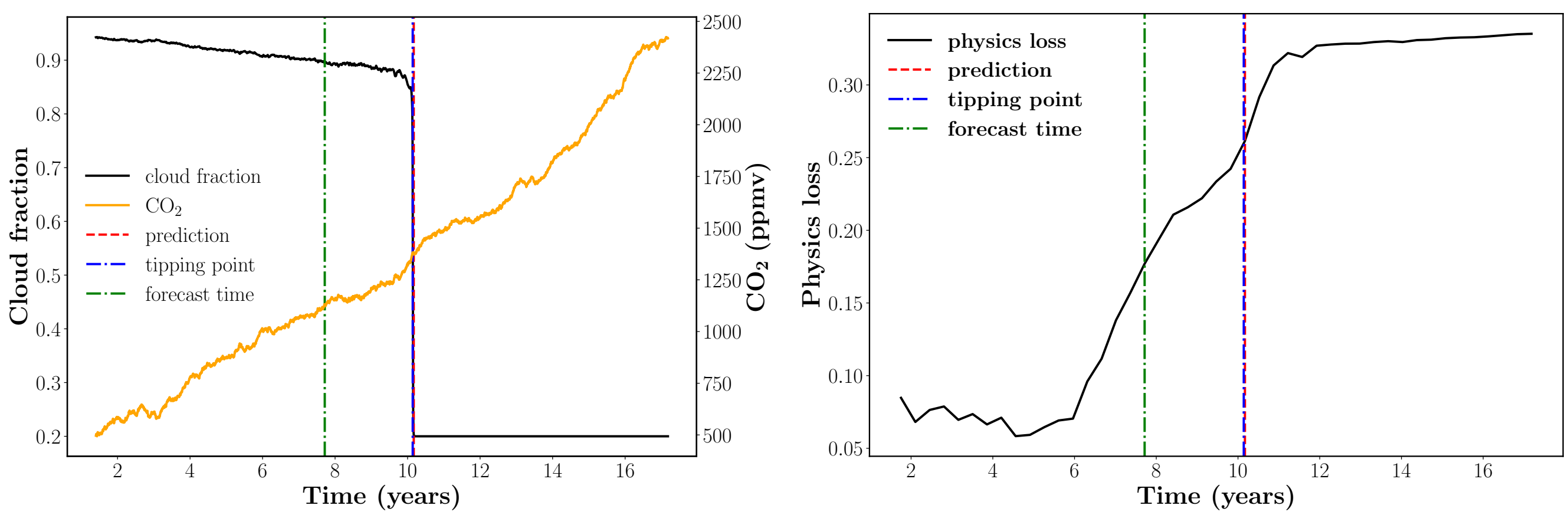

(a) Tipping point forecast w.r.t. cloud fraction

(b) Tipping point forecast w.r.t. partial physics loss

Fig. S6. Using the full ODE as the physical constraint, RNO is capable of identifying the true tipping point of the simplified cloud cover system with an error of 0.03 years, predicting $T = 2.45$ years ahead, at $\alpha = 0.08$.

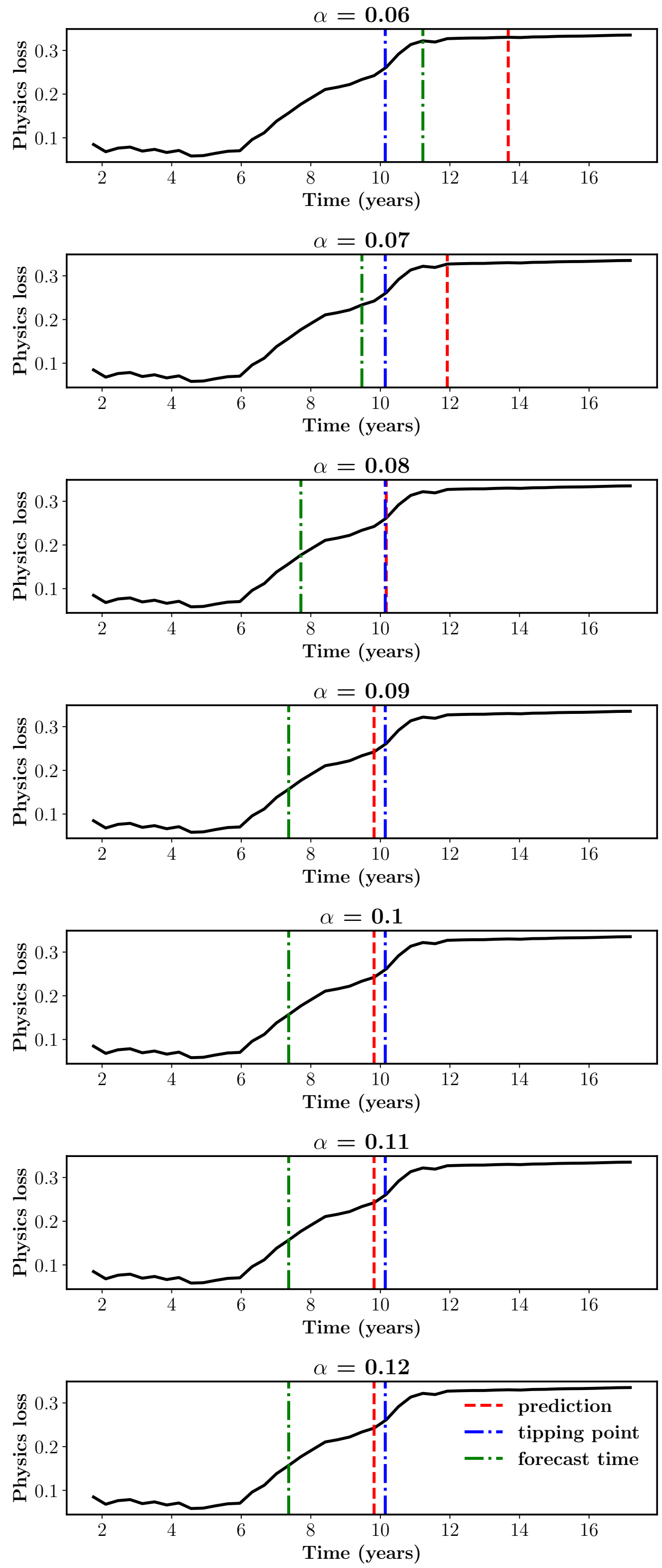

Fig. S7. Tipping point forecasting performance for RNO for various false-positive rates α in the simplified cloud cover setting. RNO achieves very low forecasting error for the majority of α. Further, RNO achieves approximately zero error for $\alpha = 0.08$.

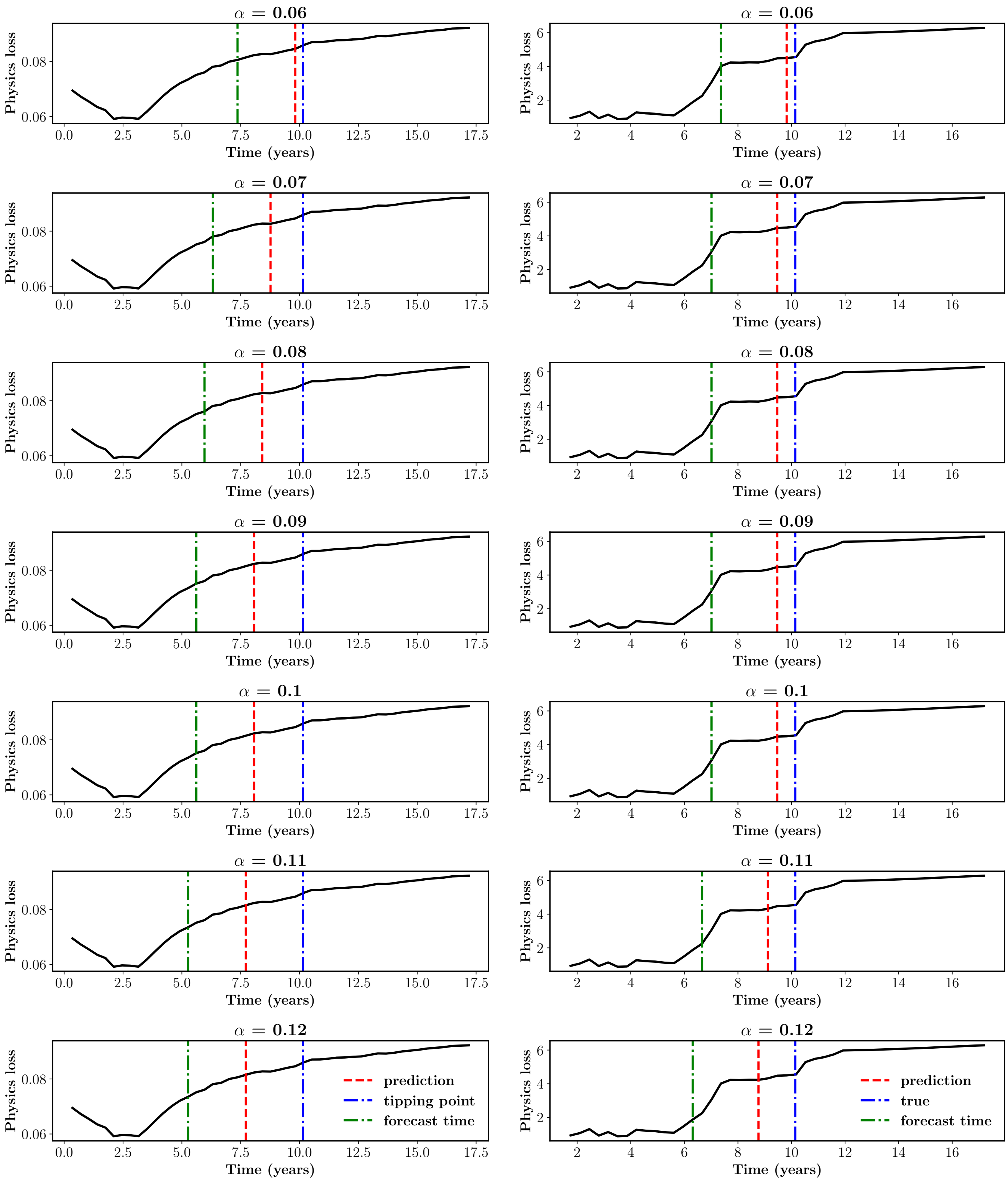

(a) MNO

(b) RNN

Fig. S8. Comparison of tipping point forecasting performance between MNO and RNN for various false-positive rates α in the simplified cloud cover setting. RNO (in Figure S7) tends to outperform MNO and RNN in tipping point forecasting error for the majority of the α.

Model	5-step	10-step	15-step	20-step	25-step
**RNN**	0.0121	0.0222	0.0326	0.0432	**0.0540**
**MNO**	0.0120	0.0220	0.0317	0.0502	0.0603
**RNO**	**0.0101**	**0.0215**	**0.0313**	**0.0411**	0.0553

Table S3. Relative L^2 errors for the Re 1000 stall transition.

Model	5-step	10-step	15-step	20-step	25-step
**RNN**	**0.0166**	**0.0269**	**0.0382**	0.0501	0.0621
**MNO**	0.0173	0.0291	0.0385	**0.0495**	**0.0611**
**RNO**	0.0180	0.0294	0.0388	0.0509	0.0631

Table S4. Relative L^2 errors for the Re 5000 wake transition.

Model	2-step	4-step	6-step	8-step
**RNN**	0.1314	0.1341	0.1369	0.1371
**MNO**	0.0105	**0.0118**	**0.0130**	**0.0146**
**RNO**	**0.0070**	0.0165	0.0250	0.0413

Table S5. Relative L^2 errors for the Re 5000 stall transition.

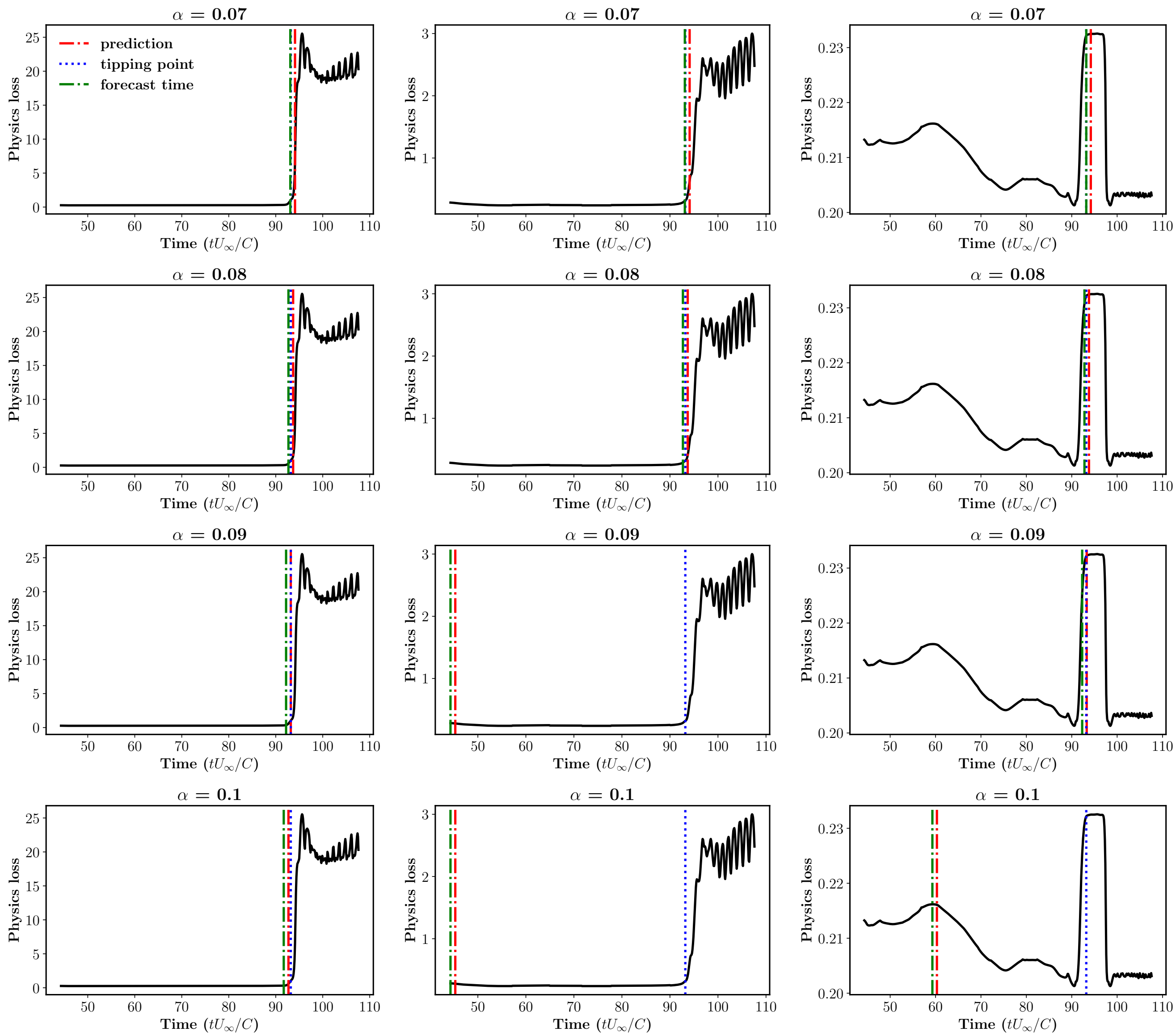

(a) RNO **(b)** MNO **(c)** RNN

Fig. S9. Comparison of Re 1000 stall tipping point forecasting performance between RNO, MNO, and RNN for various false-positive rates α. For all presented α, RNO outperforms the other two models in tipping point forecasting error.

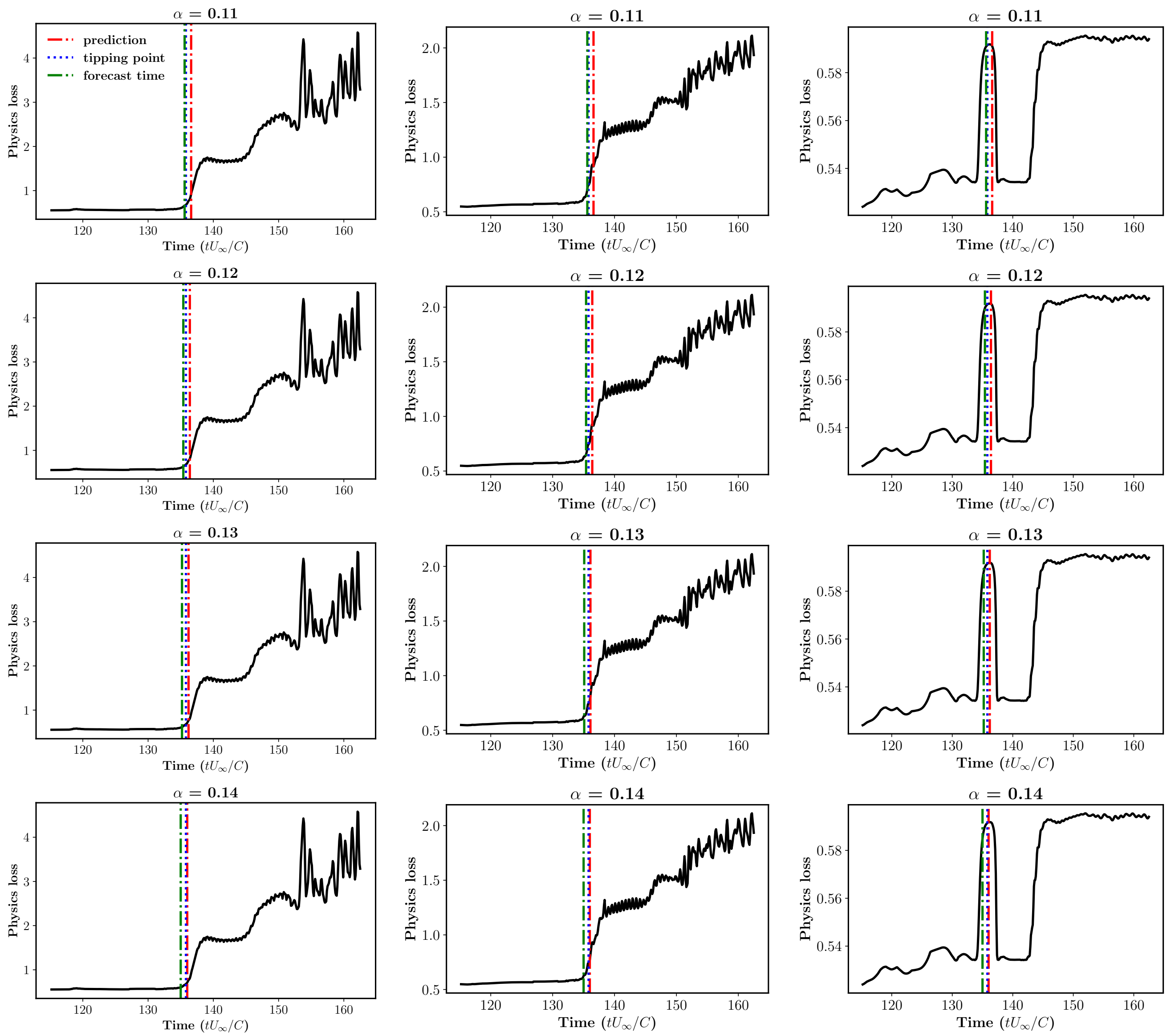

(a) RNO **(b)** MNO **(c)** RNN

Fig. S10. Comparison of Re 5000 wake tipping point forecasting performance between RNO, MNO, and RNN for various false-positive rates α.

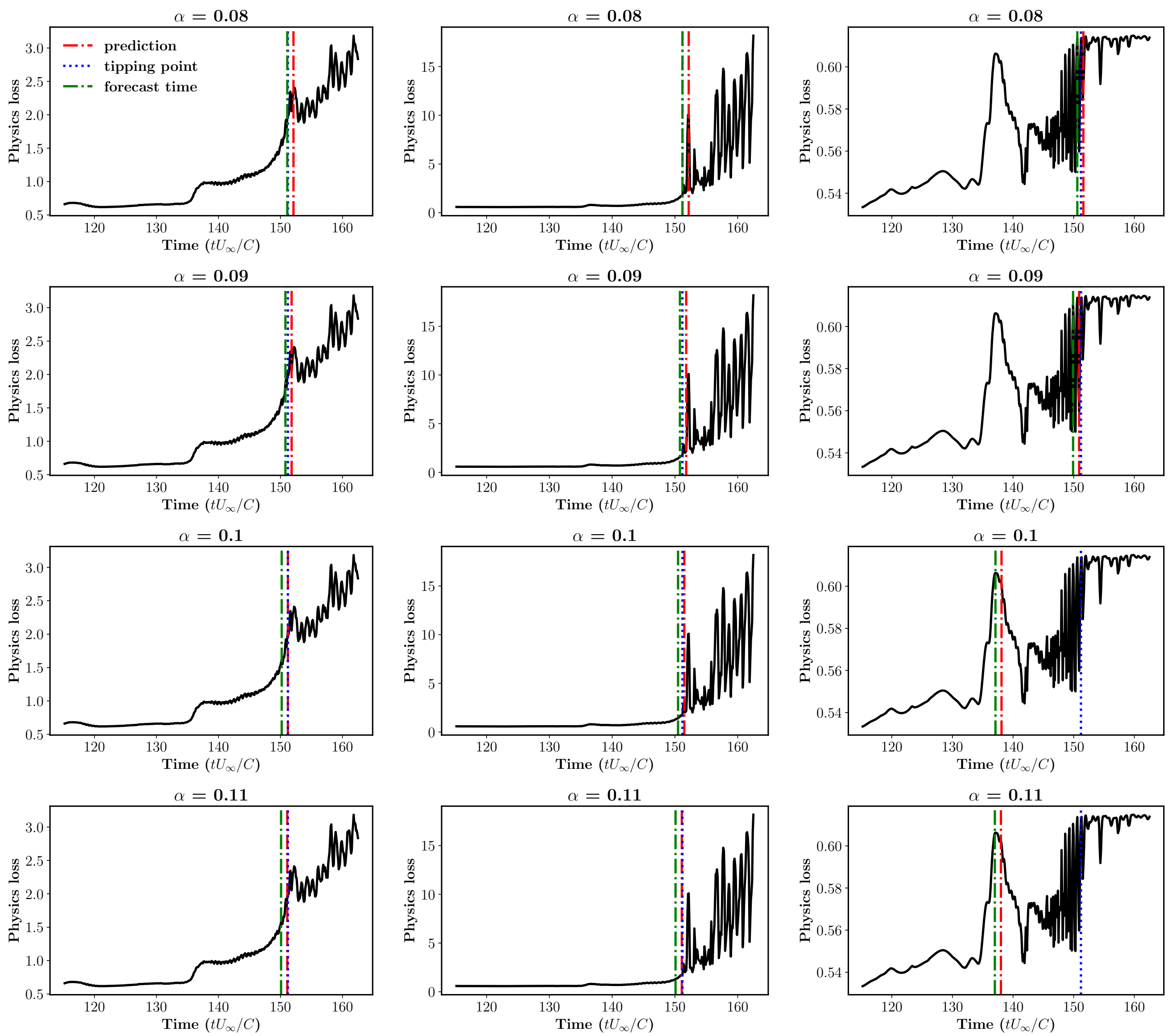

(a) RNO **(b)** MNO **(c)** RNN

Fig. S11. Comparison of Re 5000 stall tipping point forecasting performance between RNO, MNO, and RNN for various false-positive rates α.

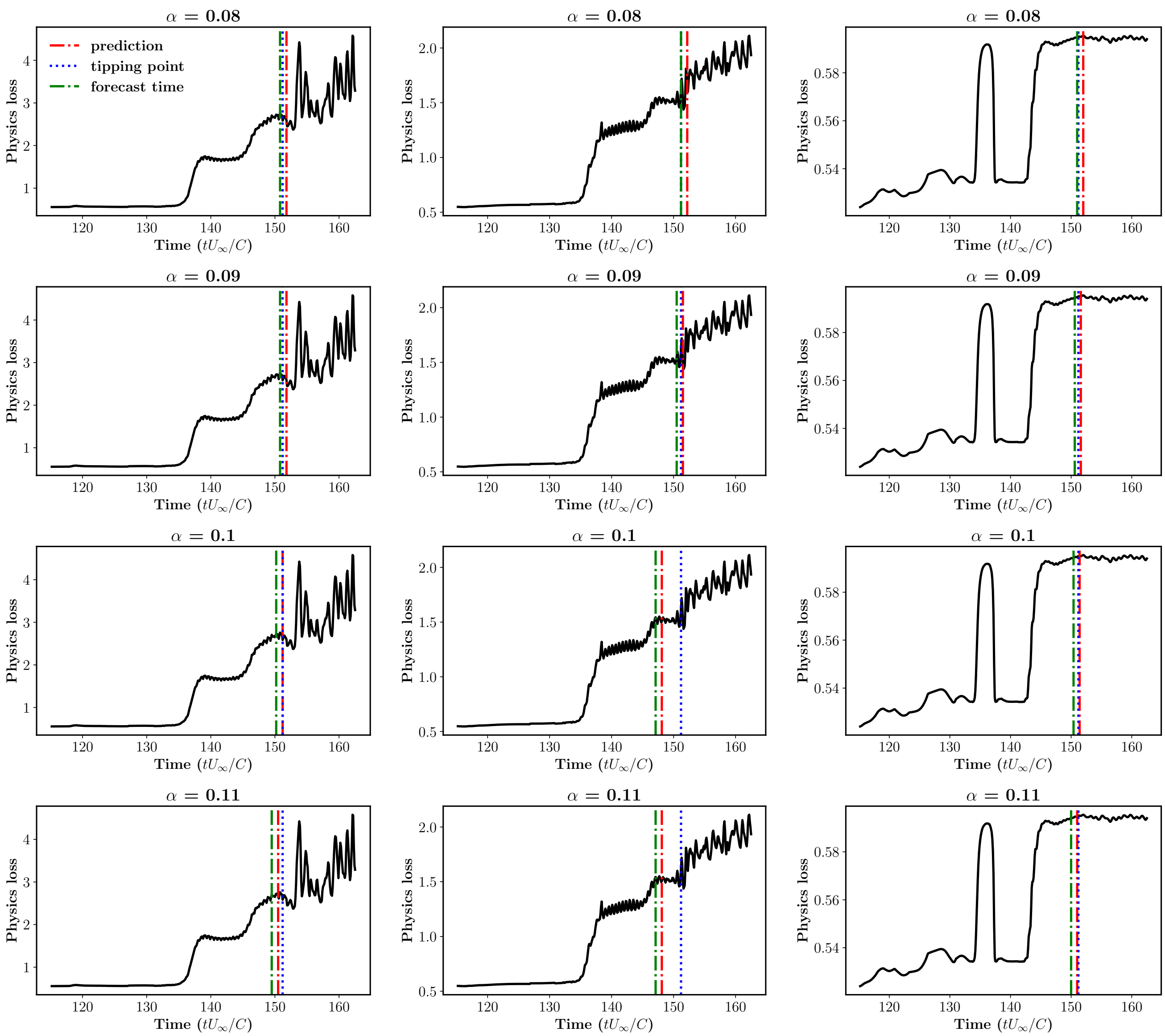

(a) RNO **(b)** MNO **(c)** RNN

Fig. S12. Comparison of Re 5000 stall tipping point forecasting performance between RNO, MNO, and RNN trained on pre-wake dynamics for various false-positive rates α.

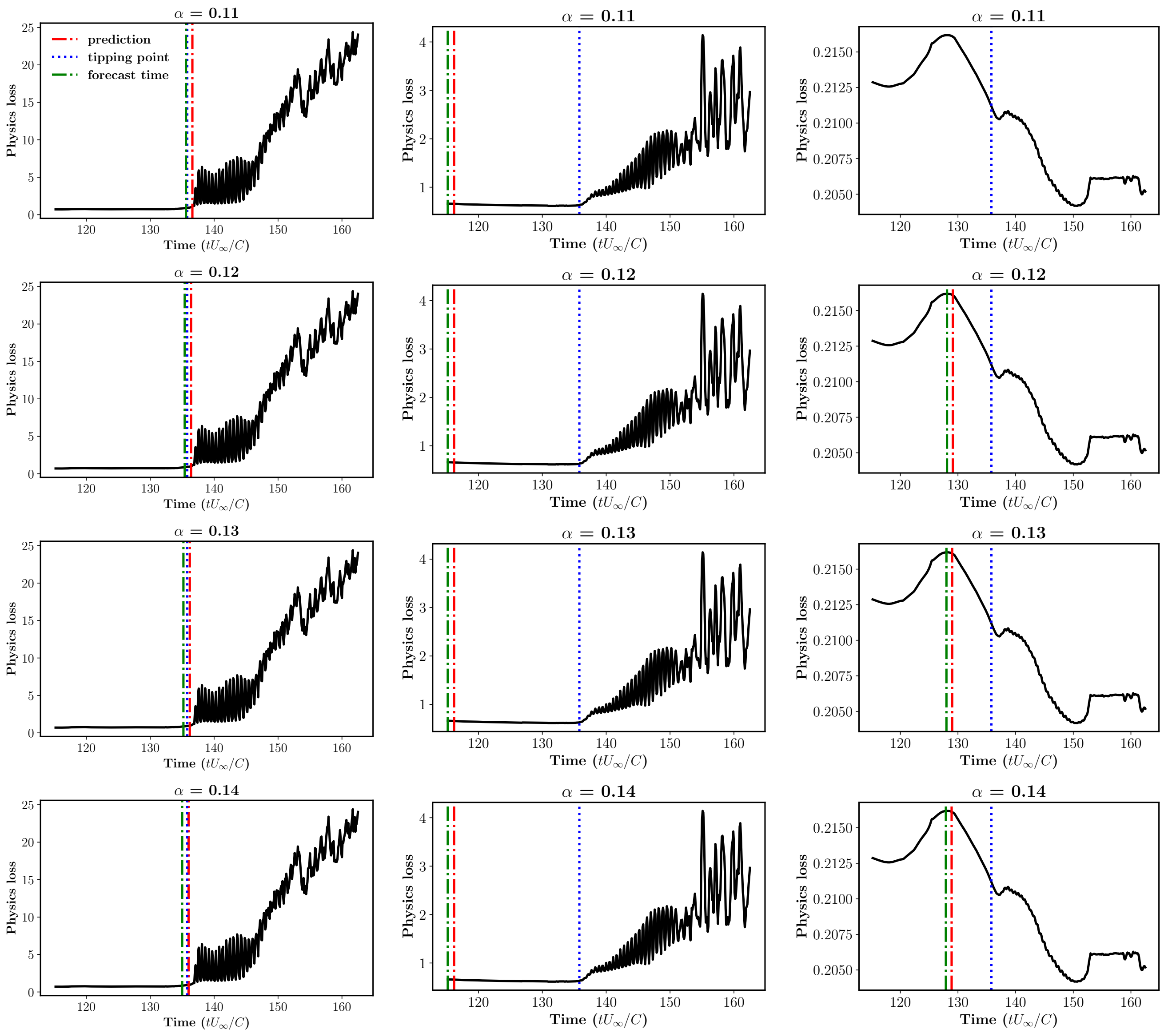

(a) RNO **(b)** MNO **(c)** RNN

Fig. S13. Comparison of Re 5000 wake tipping point forecasting performance between RNO, MNO, and RNN trained on Re 1000 pre-stall dynamics for various false-positive rates α. For $\alpha = 0.11$, the RNN predictions do not forecast any tipping point within the time horizon.

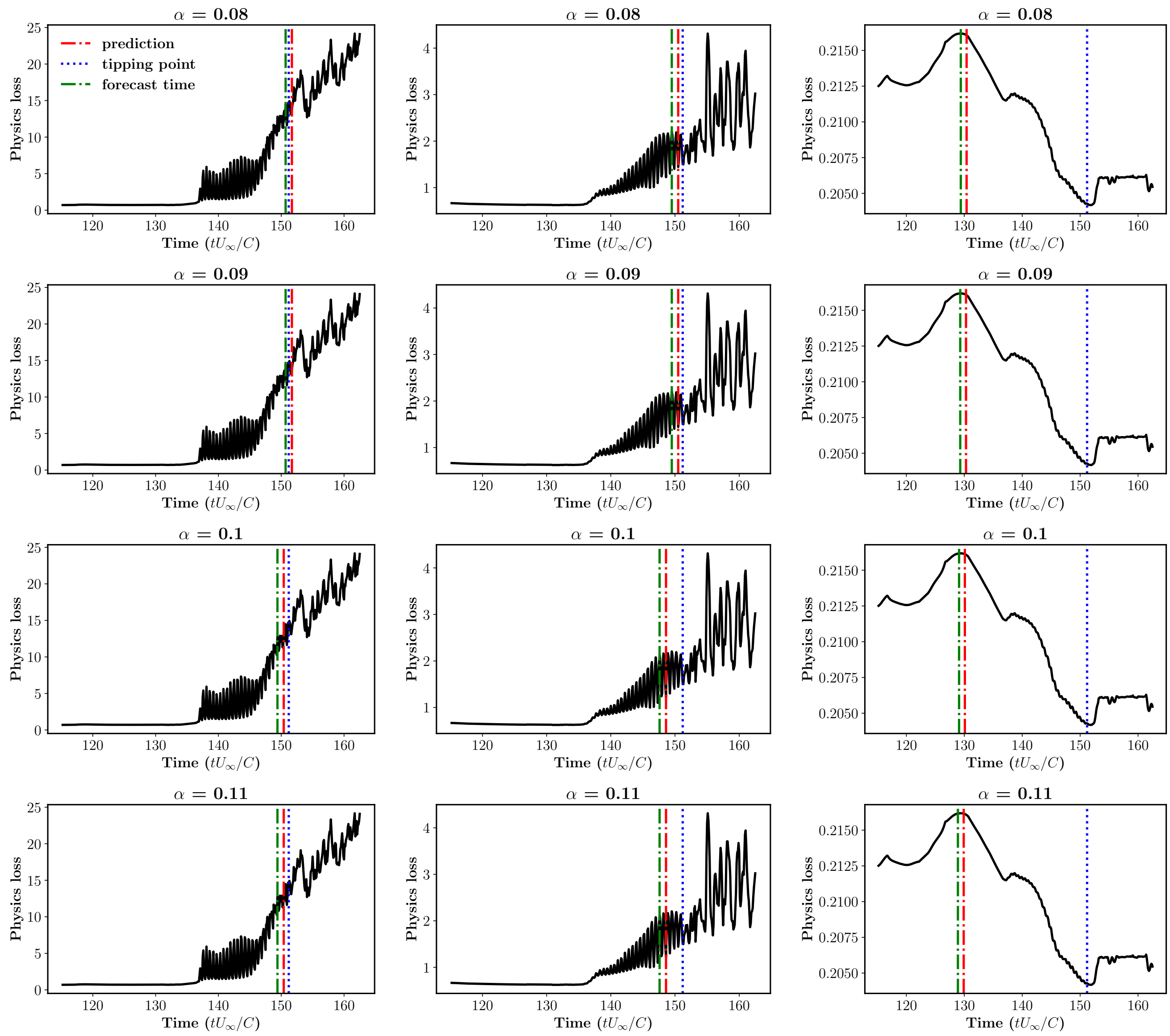

(a) RNO **(b)** MNO **(c)** RNN

Fig. S14. Comparison of Re 5000 stall tipping point forecasting performance between RNO, MNO, and RNN trained on Re 1000 pre-stall dynamics for various false-positive rates α.

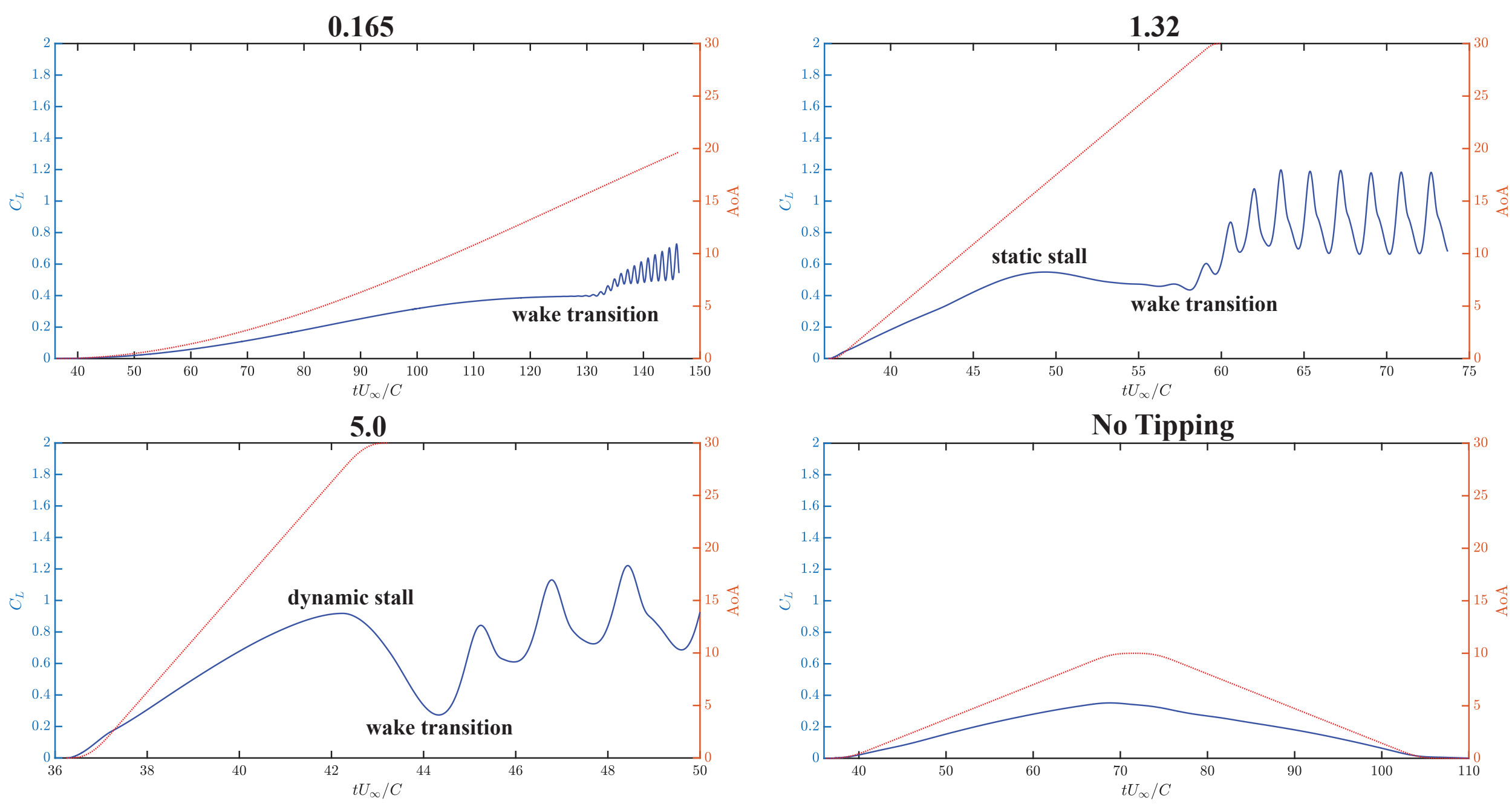

Fig. S15. Lift coefficient and angle-of-attack trajectories for different pitch rates. The lift coefficient, C_L (blue solid line), and angle of attack, AoA (red dotted line), are shown as functions of nondimensional time for cases starting from the same initial condition but with different rates of change of AoA. The rate for each case is indicated in the corresponding subplot title in units of AoA/t^*, where $t^* = tU_\infty/C$. The tipping point is labeled in each subplot.

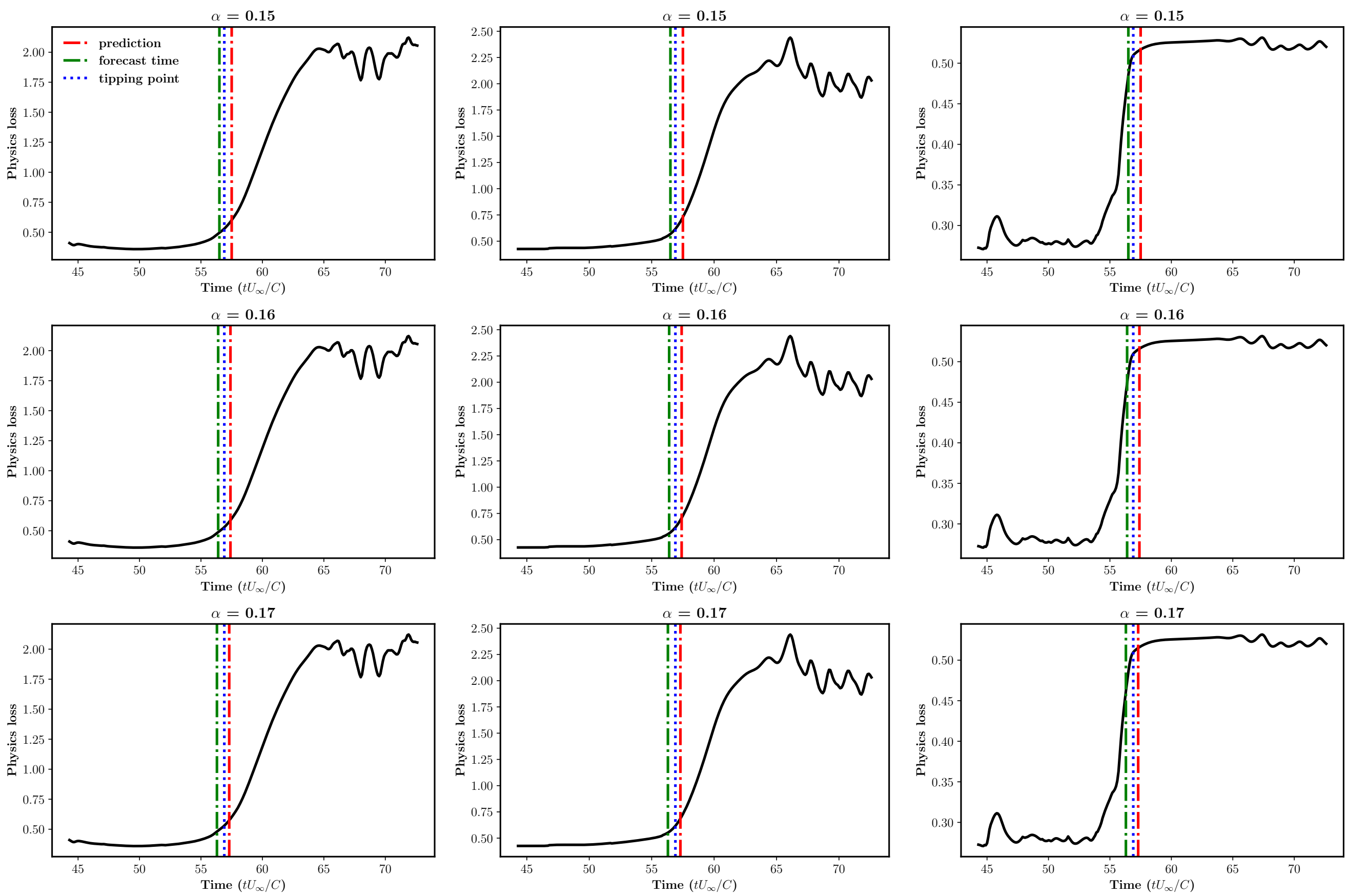

(a) RNO **(b)** MNO **(c)** RNN

Fig. S16. Comparison of tipping point forecasting performance between RNO, MNO, and RNN trained on 1.32 AoA pre-stall dynamics for various false-positive rates α.

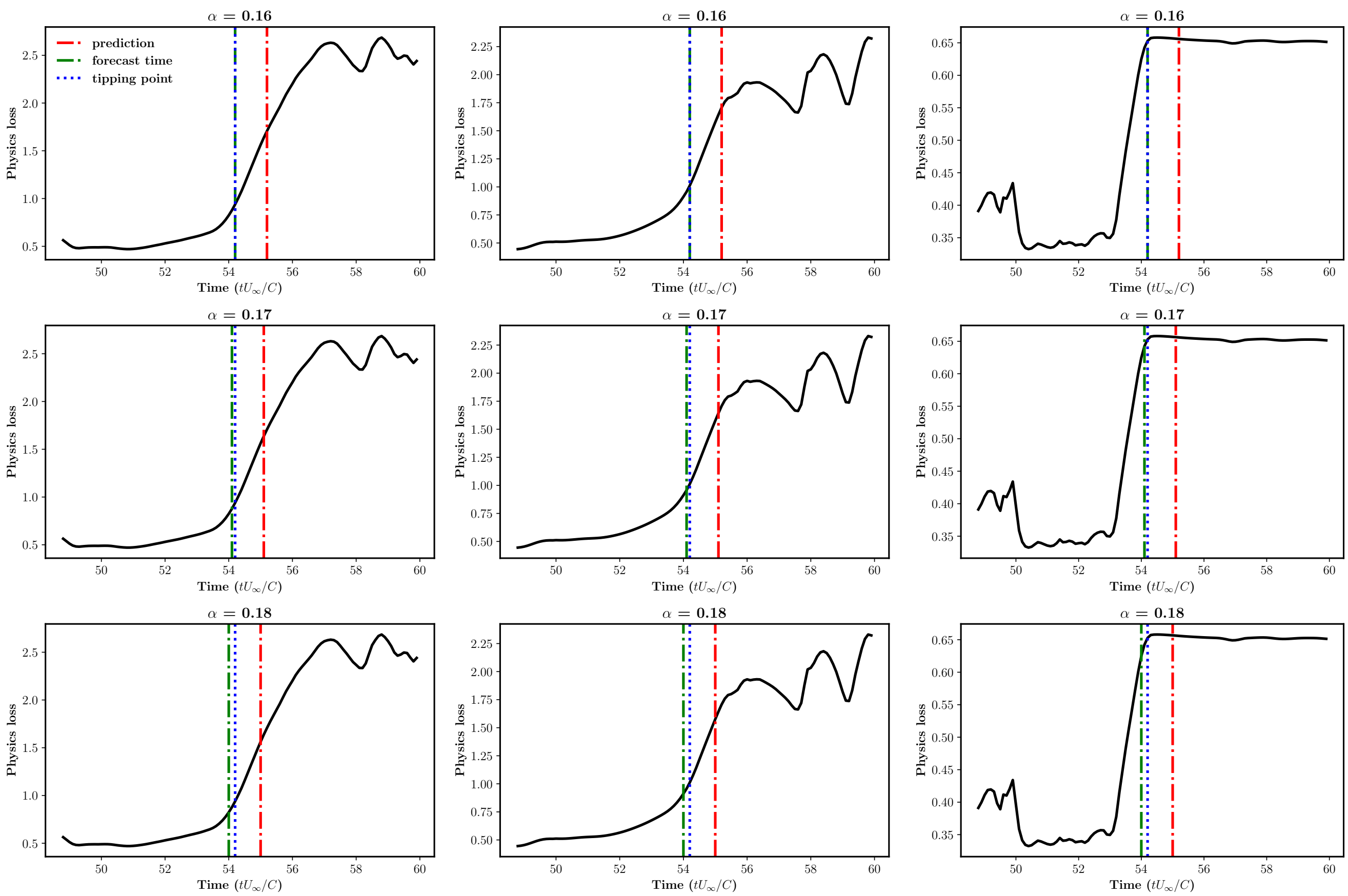

(a) RNO **(b)** MNO **(c)** RNN

Fig. S17. Comparison of tipping point forecasting performance between RNO, MNO, and RNN trained on 5.0 AoA pre-stall dynamics for various false-positive rates α.

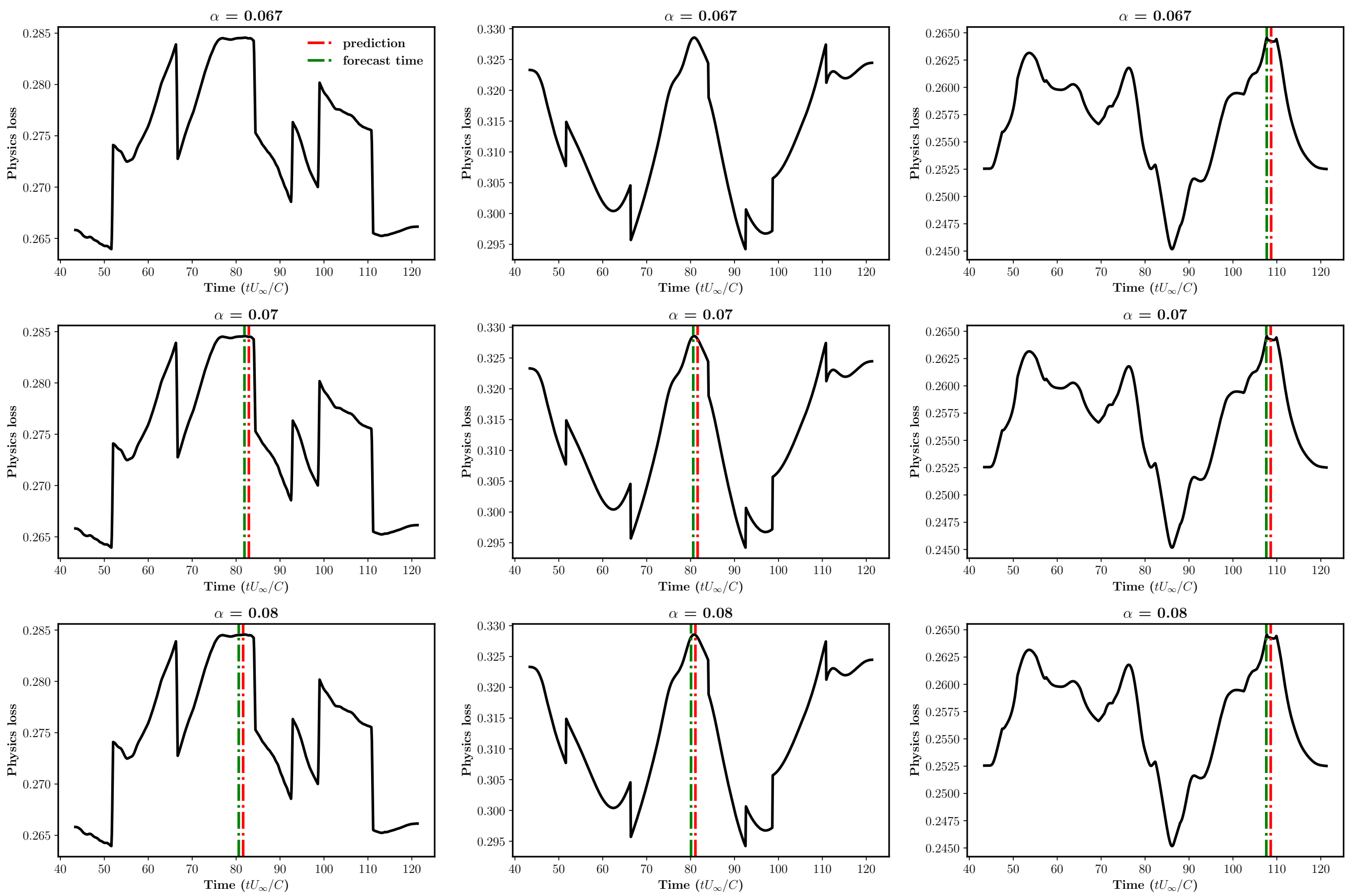

(a) RNO

(b) MNO

(c) RNN

Fig. S18. Comparison of tipping point forecasting performance between RNO, MNO, and RNN trained on non-tipping dynamics for various false-positive rates α.

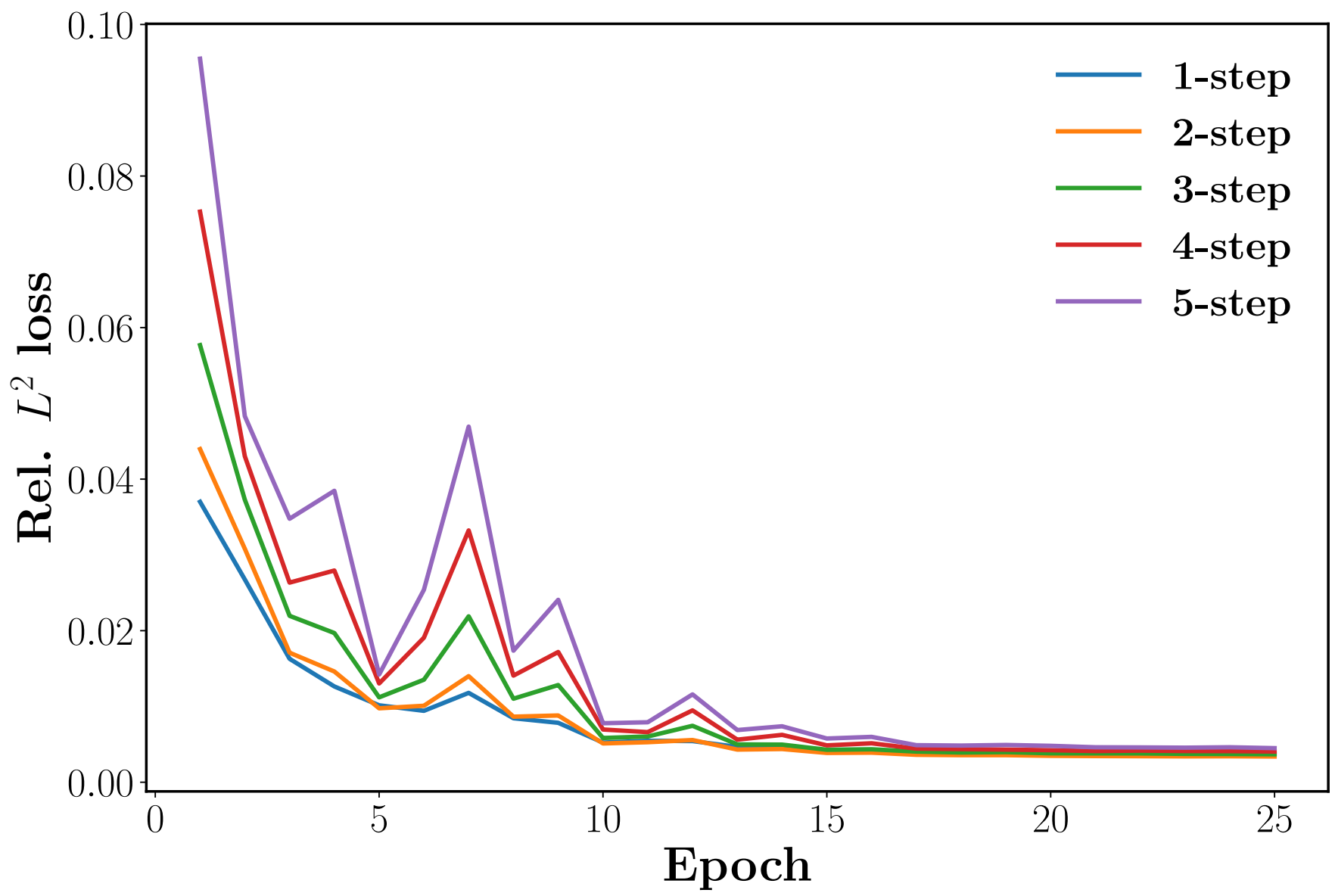

Fig. S19. Convergence plot of test relative L^2 error for RNO trained on the pre-tipping regime of the KS equation. We observe that RNO ultimately converges to near-zero error for all steps.

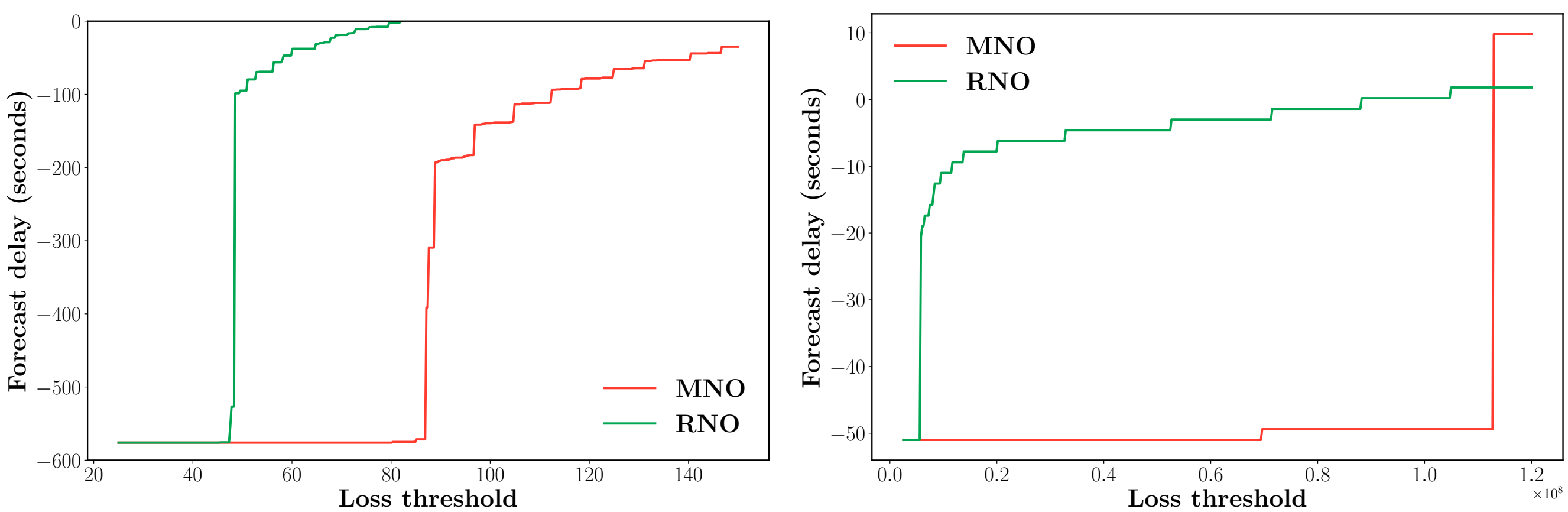

(a) (b)

Fig. S20. **(a)** Physics loss threshold comparison between RNO and MNO for non-stationary Lorenz-63 system predicting 64 seconds ahead. **(b)** Physics loss threshold comparison between proposed RNO and the state-of-the-art baseline MNO in non-stationary Kuramoto–Sivashinsky system for forecasting the tipping point 16 seconds ahead. Negative numbers correspond to tipping points predicted early. RNO consistently outperforms MNO in tipping point forecasting for a given physics loss threshold, demonstrating that RNO learns the physics of the underlying system better that MNO.

Model	1-step	2-step	4-step	8-step	16-step	32-step	64-step	128-step
RNO	**0.0053**	**0.0033**	**0.0027**	**0.0028**	**0.0068**	**0.0434**	**0.1549**	**0.2585**
RNN-8	0.0141	0.0139	0.0173	0.0150	0.0332	0.1096	0.2366	0.3434
RNN-12	0.0532	0.0594	0.0598	0.0618	0.0813	0.1433	0.2231	0.3052
RNN-1	0.0114	0.1644	0.2729	0.3546	0.4030	0.4153	0.4057	0.4006

Table S6. Relative L^2 errors on non-stationary Lorenz-63 for different n-step prediction settings and number of RNN fine-tuning steps M (denoted "RNN-M"). RNO outperforms RNN on every forecasting interval and for every RNN training setup.

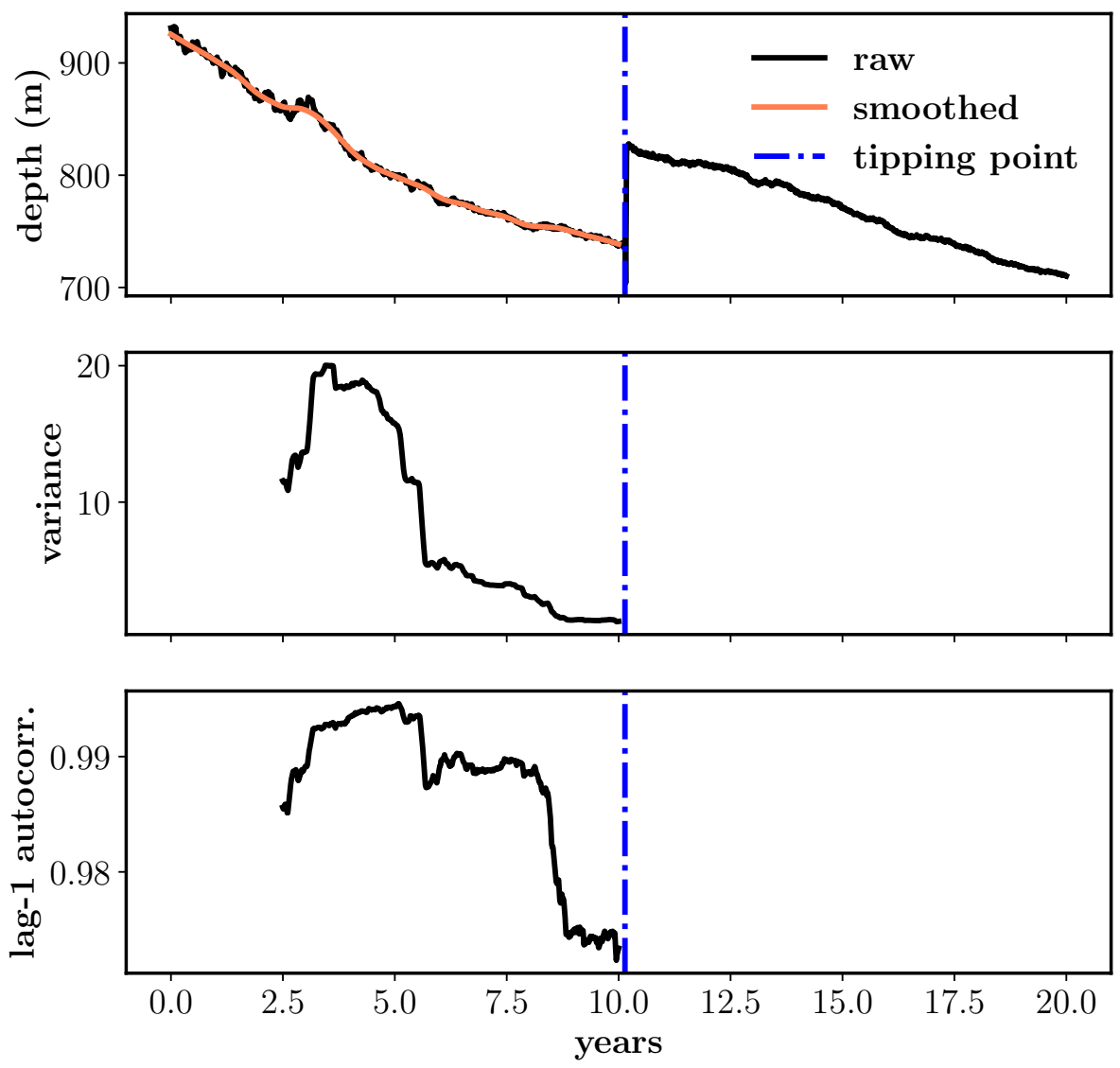

(a) Boundary layer depth

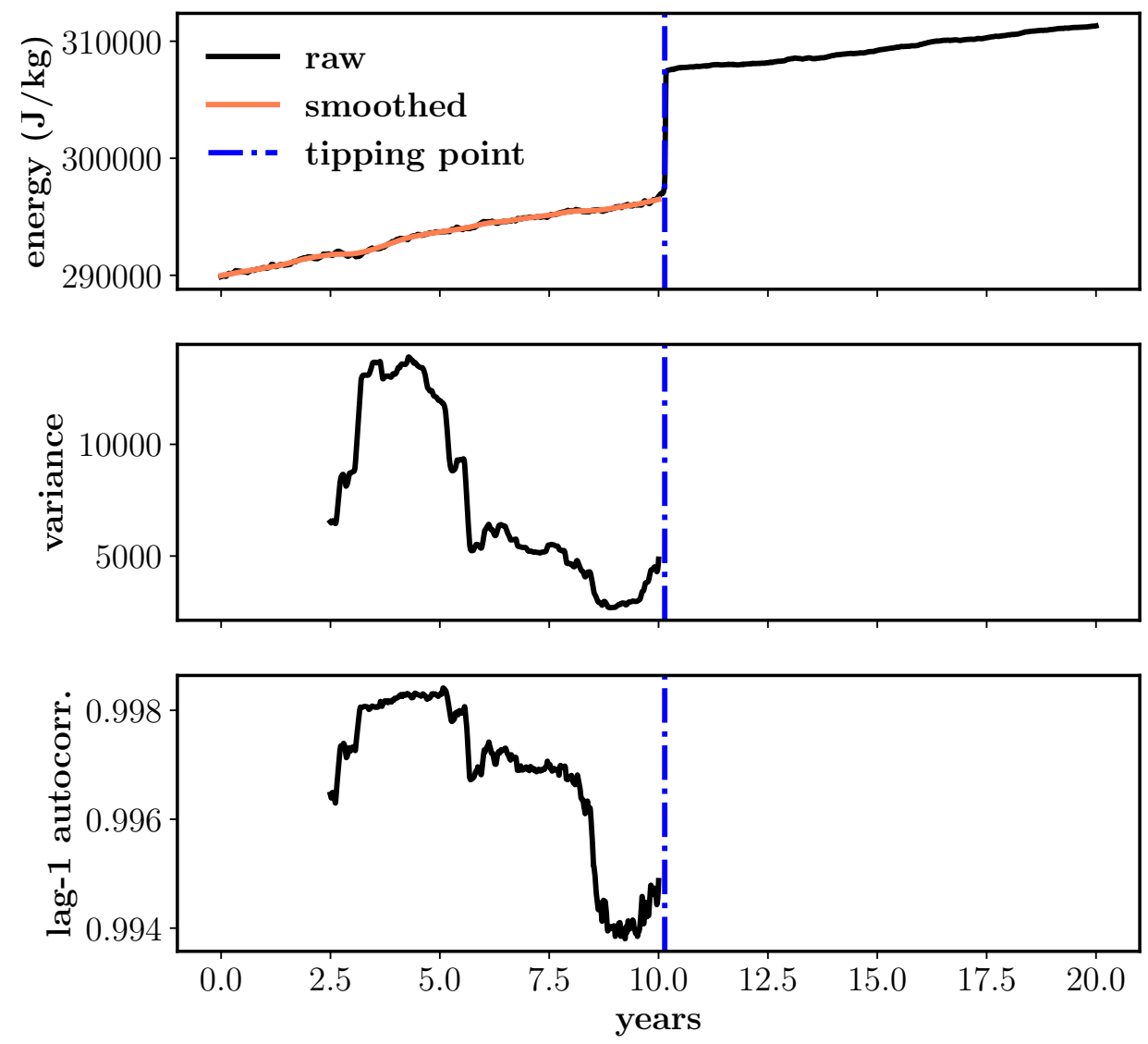

(b) Liquid water static energy

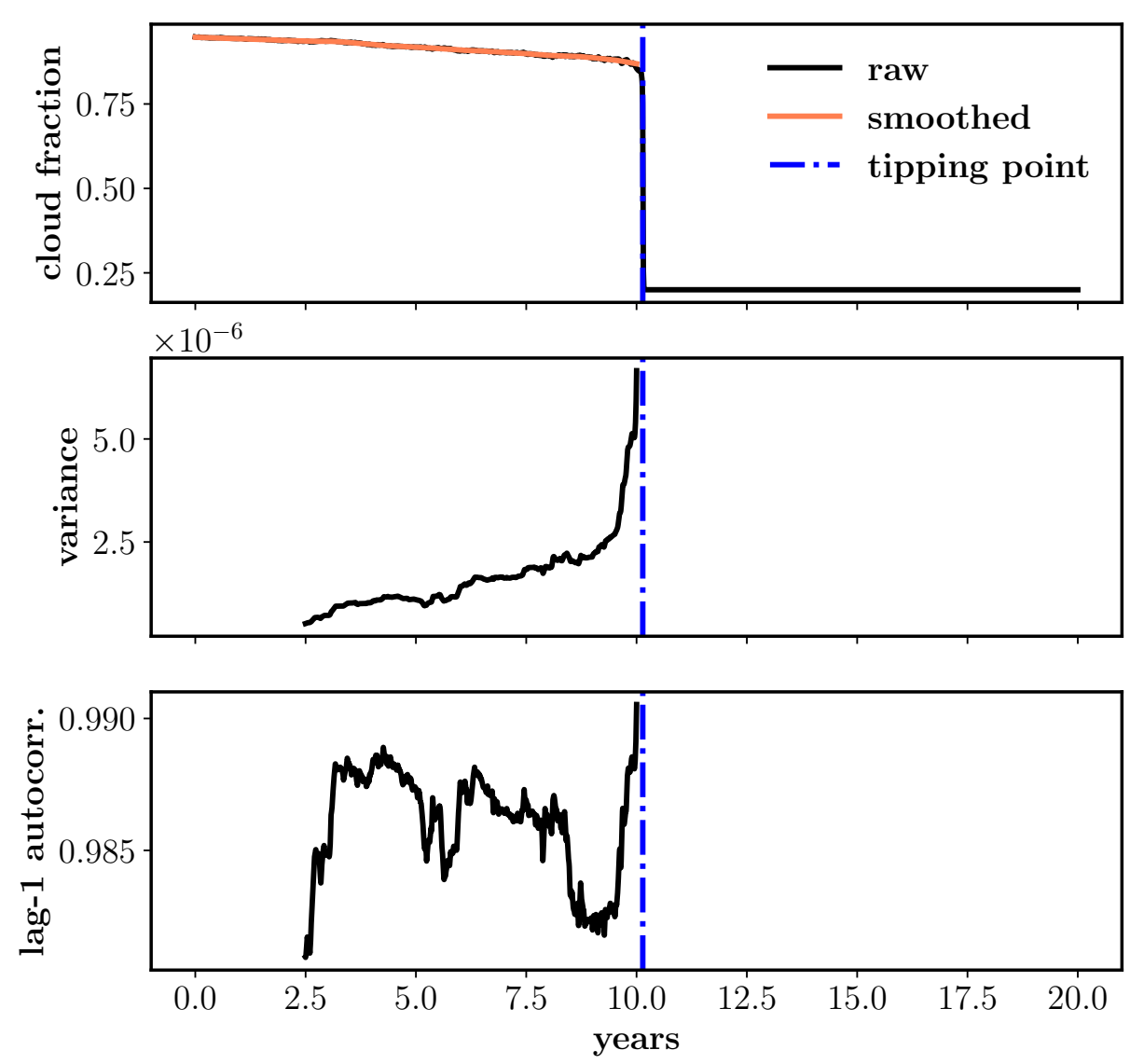

(c) Cloud fraction

Fig. S21. Variance and lag-1 autocorrelation applied as early warning signals to forecast impending tipping points in the surrogate cloud cover system. Kendall's τ values for (a) variance: -0.83, autocorrelation: -0.48, (b) variance: -0.69, autocorrelation: -0.59, and (c) variance: 0.89, autocorrelation: -0.33. Despite approaching a tipping point, autocorrelation did not tend to increase. Only the variance of cloud fraction increased.

SI Dataset S1 (airfoil_stall_2D_dataset.zip)

We release the preprocessed divergence-free trajectory data for airfoil stall at Re1000 and Re5000, as used in our experiments, and which can be found at this URL: https://zenodo.org/records/18098807.

SI Dataset S2 (Code)

We release the code repository used for our experiments and analysis, including instructions on how to generate data for the non-stationary Lorenz-63, non-stationary Kuramoto-Sivashinsky, and cloud cover experiments. The repository can be found at this URL: https://github.com/neuraloperator/tipping-point-forecast.

References

1. EN Lorenz, Deterministic nonperiodic flow. *J. atmospheric sciences* **20**, 130–141 (1963).
2. D Patel, E Ott, Using machine learning to anticipate tipping points and extrapolate to post-tipping dynamics of non-stationary dynamical systems. *arXiv preprint arXiv:2207.00521* (2022).
3. P Manneville, Y Pomeau, Intermittency and the lorenz model. *Phys. Lett. A* **75**, 1–2 (1979).
4. P Ashwin, S Wieczorek, R Vitolo, P Cox, Tipping points in open systems: bifurcation, noise-induced and rate-dependent examples in the climate system. *Philos. Transactions Royal Soc. A: Math. Phys. Eng. Sci.* **370**, 1166–1184 (2012).
5. CE Singer, T Schneider, Stratocumulus-cumulus transition explained by bulk boundary layer theory. *ESS Open Arch.* **2023** (2023).
6. CE Singer, T Schneider, Co 2 -driven stratocumulus cloud breakup in a bulk boundary layer model. *ESS Open Arch.* **2023** (2023).
7. B Stevens, Bulk boundary-layer concepts for simplified models of tropical dynamics. *Theor. Comput. Fluid Dyn.* **20**, 279–304 (2006).
8. CS Bretherton, MC Wyant, Moisture transport, lower-tropospheric stability, and decoupling of cloud-topped boundary layers. *J. Atmos. Sci.* **54**, 148–167 (1997).
9. D Chung, J Teixeira, A simple model for stratocumulus to shallow cumulus cloud transitions. *J. Clim.* **25**, 2547–2554 (2012).
10. G Cesana, AD Del Genio, H Chepfer, The cumulus and stratocumulus CloudSat-CALIPSO dataset (CASCCAD). *Earth Syst. Sci. Data* **11**, 1745–1764 (2019).
11. Y Zheng, Y Zhu, D Rosenfeld, Z Li, Climatology of cloud-top radiative cooling in marine shallow clouds. *Geophys. Res. Lett.* **48**, e2021GL094676 (2021).
12. T Schneider, CM Kaul, KG Pressel, Possible climate transitions from breakup of stratocumulus decks under greenhouse warming. *Nat. Geosci.* **12**, 163–167 (2019).
13. D You, F Ham, P Moin, Discrete conservation principles in large-eddy simulation with application to separation control over an airfoil. *Phys. Fluids* **20**, 101515 (2008).
14. HA Van der Vorst, Bi-cgstab: A fast and smoothly converging variant of bi-cg for the solution of nonsymmetric linear systems. *SIAM J. on scientific Stat. Comput.* **13**, 631–644 (1992).
15. M Germano, U Piomelli, P Moin, WH Cabot, A dynamic subgrid-scale eddy viscosity model. *Phys. Fluids A: Fluid Dyn.* **3**, 1760–1765 (1991).
16. DK Lilly, A proposed modification of the germano subgrid-scale closure method. *Phys. Fluids A: Fluid Dyn.* **4**, 633–635 (1992).
17. JD Anderson, *Computational Fluid Dynamics: The Basics with Applications.* (McGraw-Hill, New York), p. 199 (1995).
18. Z Li, et al., Learning chaotic dynamics in dissipative systems. *Adv. Neural Inf. Process. Syst.* **35**, 16768–16781 (2022).
19. K Cho, et al., Learning phrase representations using rnn encoder-decoder for statistical machine translation. *arXiv preprint arXiv:1406.1078* (2014).
20. Z Li, et al., Fourier neural operator for parametric partial differential equations. *arXiv preprint arXiv:2010.08895* (2020).
21. DP Kingma, J Ba, Adam: A method for stochastic optimization. *arXiv preprint arXiv:1412.6980* (2014).
22. TM Bury, et al., Deep learning for early warning signals of tipping points. *Proc. Natl. Acad. Sci.* **118**, e2106140118 (2021).
23. D Patel, D Canaday, M Girvan, A Pomerance, E Ott, Using machine learning to predict statistical properties of non-stationary dynamical processes: System climate, regime transitions, and the effect of stochasticity. *Chaos: An Interdiscip. J. Nonlinear Sci.* **31**, 033149 (2021).
24. LW Kong, HW Fan, C Grebogi, YC Lai, Machine learning prediction of critical transition and system collapse. *Phys. Rev. Res.* **3**, 013090 (2021).
25. SH Lim, L Theo Giorgini, W Moon, JS Wettlaufer, Predicting critical transitions in multiscale dynamical systems using reservoir computing. *Chaos: An Interdiscip. J. Nonlinear Sci.* **30**, 123126 (2020).
26. X Li, et al., Tipping point detection using reservoir computing. *Research* **6**, 0174 (2023).
27. S Deb, S Sidheekh, CF Clements, NC Krishnan, PS Dutta, Machine learning methods trained on simple models can predict critical transitions in complex natural systems. *Royal Soc. Open Sci.* **9**, 211475 (2022).
28. J Sleeman, et al., A generative adversarial network for climate tipping point discovery (tip-gan). *arXiv preprint arXiv:2302.10274* (2023).

29. Y Huang, S Bathiany, P Ashwin, N Boers, Deep learning for predicting rate-induced tipping. *Nat. Mach. Intell.* pp. 1–10 (2024).
30. S Panahi, et al., Machine learning prediction of tipping in complex dynamical systems. *Phys. Rev. Res.* **6**, 043194 (2024).
31. G Fabiani, et al., Task-oriented machine learning surrogates for tipping points of agent-based models. *Nat. communications* **15**, 4117 (2024).
32. S Panahi, LW Kong, B Glaz, M Haile, YC Lai, Unsupervised learning for anticipating critical transitions. *Phys. Rev. Lett.* **136**, 077301 (2026).
33. M Scheffer, et al., Early-warning signals for critical transitions. *Nature* **461**, 53–59 (2009).
34. V Dakos, et al., Methods for detecting early warnings of critical transitions in time series illustrated using simulated ecological data. *PloS one* **7**, e41010 (2012).
35. TM Bury, ewstools: A python package for early warning signals of bifurcations in time series data. *J. Open Source Softw.* **8**, 5038 (2023).
36. A Tran, A Mathews, L Xie, CS Ong, Factorized fourier neural operators. *arXiv preprint arXiv:2111.13802* (2021).
37. C White, et al., Speeding up fourier neural operators via mixed precision. *arXiv preprint arXiv:2307.15034* (2023).
38. J Pathak, et al., Fourcastnet: A global data-driven high-resolution weather model using adaptive fourier neural operators. *arXiv preprint arXiv:2202.11214* (2022).
